# Health-related quality of life in adult patients with asthma according to asthma control and severity: A systematic review and meta-analysis

**DOI:** 10.3389/fphar.2022.908837

**Published:** 2022-11-21

**Authors:** Byeong-Chan Oh, Ju-Eun Lee, Jin Hyun Nam, Ji-Yoon Hong, Sun-Hong Kwon, Eui-Kyung Lee

**Affiliations:** ^1^ School of Pharmacy, Sungkyunkwan University, Suwon, South Korea; ^2^ Divison of Big Data Science, Korea University Sejong Campus, Sejong, South Korea; ^3^ Health Insurance Research Institute, National Health Insurance Service, Wonju, South Korea

**Keywords:** asthma, quality of life, utility, systematic review, meta-analysis

## Abstract

**Background:** The utility values are increasingly being used in economic evaluations and health policy decision making. This study aims to conduct a systematic literature review and meta-analysis of the utility values for asthma, particularly with respect to severity and asthma control.

**Materials and methods:** A literature search was conducted using the MEDLINE, Embase, and Cochrane Central Register of Controlled Trials databases for studies published until July, 2020, reporting the utilities of adult asthma. We extracted utility values derived by nine indirect and four direct utility instruments. Meta-analyses were performed for each utility instrument according to health states based on the level of asthma control and severity.

**Results:** Fifty-two eligible studies were included in our systematic review, of which forty studies were used in the meta-analyses. Among the 13 utility instruments, the most used was EQ-5D-3L, whereas EQ-5D-5L showed the narrowest 95% confidence interval (95% CI, 0.83–0.86) of pooled utility. The pooled utility of asthma declined with worsening control levels and severity. The pooled utility value of EQ-5D-3L was 0.72 (95% CI, 0.63–0.80) for uncontrolled, 0.82 (95% CI, 0.75–0.88) for partly controlled, and 0.87 (95% CI, 0.84–0.90) for well-controlled asthma.

**Conclusion:** Our study shows that EQ-5D-3L and EQ-5D-5L are appropriate for economic evaluations in terms of availability and variability of information, respectively. Asthma patients had poorer utility values with worsened severity and level of asthma control. This study will be useful for health economists conducting economic evaluations of asthma treatments.

## 1 Introduction

Asthma is the most common chronic disease, with patients suffering from it worldwide (Asthma Fact sheet, 2017). Asthma causes symptoms, such as shortness of breath, chest tightness, coughing, and wheezing attacks. Monitoring these symptoms is essential for disease management. The Global Initiative for Asthma (GINA) guidelines provide two different assessment criteria based on severity (mild, moderate, or severe) and level of asthma control (well-controlled, partly controlled, or uncontrolled) (Global Initiative for Asthma, 2021). Despite the global decline in asthma mortality with the increased use of inhaled corticosteroids in recent years, asthma continues to cause considerable disability and deteriorates the quality of life of patients (Papi et al., 2018). Asthma places financial burden on patients and the society, including the costs of controlling symptoms, preventing exacerbation, absenteeism, and mortality. This burden is evident from the fact that the total cost of asthma to society in 2013 was $81.9 billion (Nurmagambetov et al., 2018).

Ideally, all treatments should be available for patients; however, decision makers must also consider the scarcity of available resources. Therefore, economic evaluations have been used to obtain the best treatment for the financial investments made by health care systems (Gold et al., 1996). Economic evaluations need to estimate quality-adjusted life years (Kim et al., 2018), based upon health-state utility values (HSUVs) and the length of life gained (Richardson, 1994). Economic evaluations of asthma that reflect clinical reality require the utility values according to the level of asthma control (Gerzeli et al., 2012; Ismaila et al., 2014; Willson et al., 2014). Guidelines and clinical situations are focusing on classification by control level rather than by severity, since assessment by control level considers both the current state of the patient and the risk of future adverse effects (National Asthma Education and Prevention Program, 2007; Global Initiative for Asthma, 2021).

Previously, Einarson et al. (Einarson et al., 2015) summarized utility values of asthma and chronic obstructive pulmonary disease from studies published until 2014, according to severity. They also presented a summary of studies reporting utility values as per control level, according to a broad definition of control level. Costa et al. (Costa et al., 2019) performed a meta-analysis of quality of life according to degree of asthma control, however, this analysis included only pediatric asthma patients and their caregivers. Recently, Afshari et al. (Afshari et al., 2021) conducted a systematic review and meta-analysis of EQ-5D-5-level version (EQ-5D-5L) utility values in asthma according to level of control. However, the review only included EQ-5D-5L. Given this state of the evidence, there is a need to update the available utility values in adult asthma patients including various utility instruments. Also, to the best of our knowledge, there is no meta-analysis according to the level of asthma control in adults except for a study using EQ-5D-5L.

Therefore, we aimed to provide a comprehensive summary of the available utility values in asthma according to both severity and level of control through a systematic literature review and meta-analysis.

## 2 Methods

The study protocol was prospectively registered in the PROSPERO database (reference number: CRD42021246572). This systematic review was conducted and reported in accordance with the Preferred Reporting Items for Systematic Review and Meta-Analyses (PRISMA) statement (Liberati et al., 2009).

### 2.1 Search strategy

A systematic search was conducted using MEDLINE (*via* PubMed), Embase, and Cochrane Central Register of Controlled Trials (CENTRAL) databases in July, 2020. The search strategy included Medical Subject Headings (MeSH), Embase subject headings (Emtree), and text words related to asthma, quality of life, and utility instruments. Our search strategies for the three databases are shown in Supplementary Tables S2.1–S2.3.

### 2.2 Inclusion and exclusion criteria

The inclusion criteria were as follows: studies reporting the utility of asthma in adults using EQ-5D-3-level version (EQ-5D-3L) (Brooks, 1996; Herdman et al., 2011), EQ-5D-5L (Herdman et al., 2011), health utilities index (HUI)-2 (Mo et al., 2004), HUI-3, short form-6D (SF-6D) (Brazier et al., 2002), asthma symptom utility index (ASUI) (Bime et al., 2012), asthma quality of life utility index (AQL-5D) (Sullivan et al., 2016), 15D (Sintonen, 2001), quality of well-being (QWB) (Kaplan and Bush, 1982; Kaplan et al., 1989), EuroQol-visual analog scale (EQ-VAS), visual analog scale (VAS) (Torrance et al., 2001), standard gamble (SG) (Torrance, 1976), and time trade-off (TTO) (Lugnér and Krabbe, 2020). Secondary research was included only if unpublished results from the original research were cited. Only full-text articles in English were included in this study. Conference abstracts were not considered because they frequently report incomplete or non-peer-reviewed data. Studies using mapping algorithms to calculate preference-based health utilities were excluded. We did not apply date limits or study design restrictions because studies reporting utility values do not fall into a particular study design. Studies that were not clinically or methodologically comparable were excluded, such as studies that reported utility values of asthma patients with intervention (e.g., digital asthma self-management intervention) or focused on specific type of asthma (e.g., with a blood eosinophil count≥400 cells/㎕). The detailed inclusion and exclusion criteria are summarized in Supplementary Table S1 and the citations of excluded full texts are presented in Supplementary Table S3.

### 2.3 Screening and data extraction

Titles and abstracts were reviewed for eligibility as per the inclusion criteria. The full texts remaining at this stage were further screened against the inclusion criteria. These steps were performed by two reviewers: one who conducted the initial screening, and another who validated the decisions. Discrepancies between the reviewers were resolved by consensus, and if the disagreement persisted, a third reviewer made the final decision.

Data extraction was performed by two reviewers using a standardized data extraction template in Microsoft Excel (version 2016; Microsoft, Redmond, WA, United States). One reviewer performed the initial extraction and another crosschecked the extracted data. The following data were extracted: study characteristics (year of publication, geographic location, and study design), patient demographics (age, sex, asthma severity, and level of asthma control), sample size, utility instrument used, and utility values. When utility values were measured multiple times during the follow-up period, the first measurement or baseline utility was extracted to use comparable utilities not confounded by further treatment.

### 2.4 Quality assessment

To our knowledge, there are no agreed-upon reporting standards for HSUV studies. Therefore, the quality of the included studies was evaluated using the criteria framework set described by Papaionannou et al. (Papaioannou et al., 2013), which was used in previous studies (Meregaglia and Cairns, 2017; Saeed et al., 2020; Szabo et al., 2020). The criteria were as follows: 1) sample size ≥100; 2) description of respondent selection and recruitment; 3) description of inclusion/exclusion criteria; 4) response rate ≥60%; 5) reporting of the amount and reasons for loss to follow-up; 6) reporting of the level of missing data and methods to handle the issue; and 7) appropriateness of the measure (based on the judgment of the review authors).

### 2.5 Data synthesis

Data synthesis was conducted in two parts. First, meta-analyses were performed on the general asthma utility values that did not classify asthma according to severity or level of control. All meta-analyses were stratified by utility instruments: EQ-5D-3L, EQ-5D-5L, HUI-3, HUI-2, SF-6D, ASUI, AQL-5D, 15D, QWB, EQ-VAS, VAS, SG, TTO. Second, meta-analyses were performed with the studies reporting utility values categorized into health states based on asthma severity (intermittent, mild, moderate, severe) and level of control (well-controlled, partly controlled, uncontrolled) to reduce heterogeneity. They were performed using EQ-5D-3L, EQ-5D-5L, SF-6D, and HUI-3, which are the most frequently mentioned instruments in pharmacoeconomic guidelines (Kennedy-Martin et al., 2020), and ASUI and AQL-5D, which are disease-specific instruments. The criteria for judging severity or level of control were not limited.

The literature used for meta-analyses differs in study design, therefore, the DerSimonian-Laird random effects model weighted by inverse squared standard error was used to incorporate the between-study heterogeneity (Laird and Mosteller, 1990). Standard deviation was calculated using the method presented in the Cochrane Handbook through the confidence interval (CI) and standard error if it was unreported in the literature (Higgins, 2011). Studies that did not report standard deviation, CI, and standard error were excluded from the meta-analyses. Tests for heterogeneity were performed using Higgin’s I^2^ statistic.

Publication bias was assessed using funnel plots and Egger’s regression test for meta-analysis including more than 10 studies (Egger et al., 1997; Higgins, 2011). The sensitivity analyses were performed to assess the impact of excluding studies that did not explicitly report the control-level criteria and to determine the influential studies using the leave-one-out method (Viechtbauer and Cheung, 2010). All analyses were performed in R version 4.0.3 (The R Foundation for Statistical Computing, Vienna, Austria) using the “meta” and “metafor” packages (Schwarzer, 2007; Viechtbauer, 2010).

## 3 Results

### 3.1 Study selection

**FIGURE 1:**
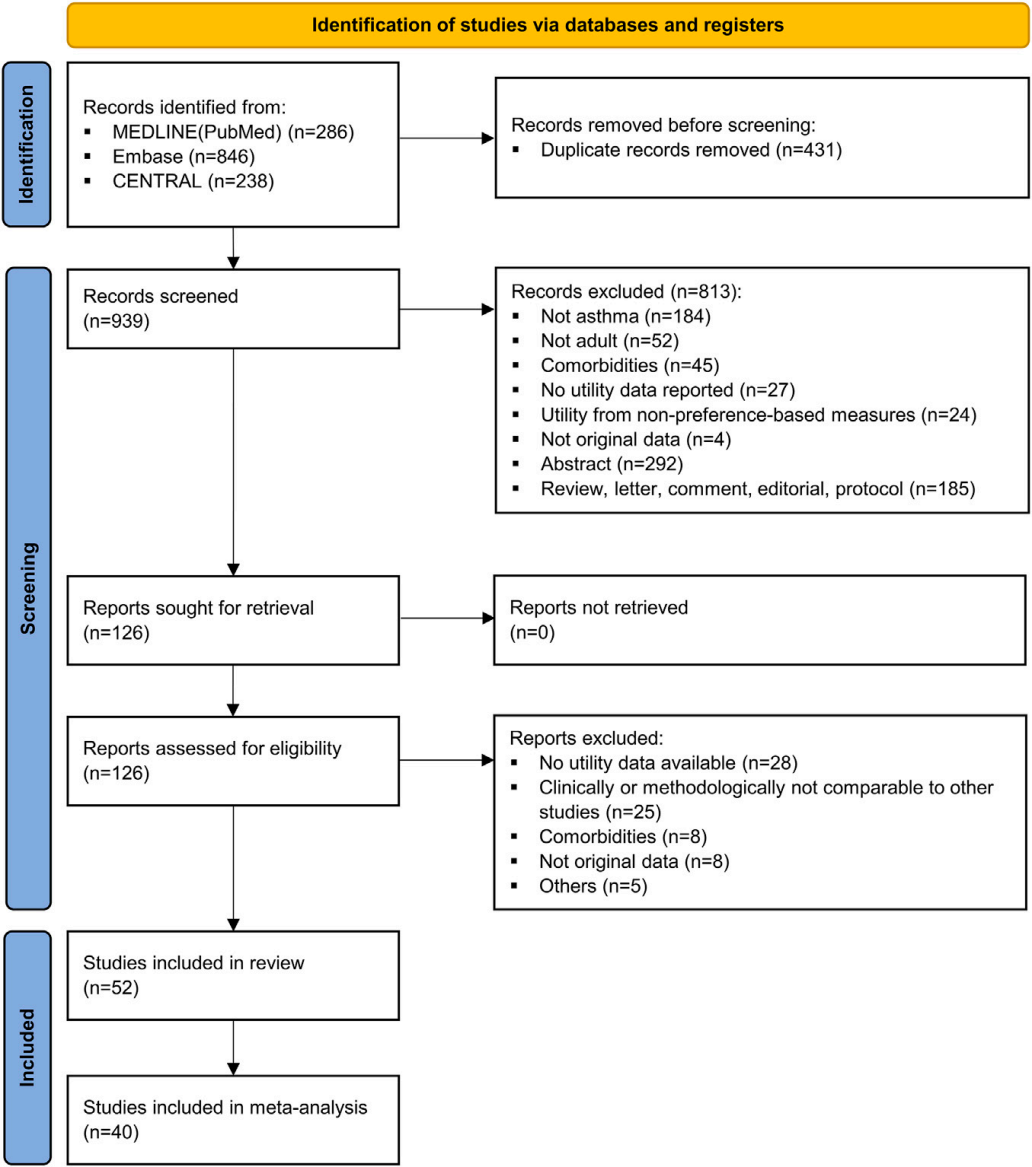
PRISMA flow diagram of study selection. CENTRAL, Cochrane Central Register of Controlled Trials; PRISMA, Preferred Reporting Items for Systematic Review and Meta-Analyses.

After removing duplicates, 939 studies were identified, of which 52 studies met the criteria (see Figure 1 for this process, and see Supplementary Table S3 for the reasons for the excluded studies) (Rutten-van Mölken et al., 1995; Blumenschein and Johannesson, 1998; Revicki et al., 1998; Mittmann et al., 1999; Burström et al., 2001; Juniper et al., 2001; Mittmann et al., 2001; Meszaros et al., 2003; Moy et al., 2004; Smith et al., 2004; Szende et al., 2004; Lubetkin et al., 2005; Flood et al., 2006; Aburuz et al., 2007; Chen et al., 2007; Willems et al., 2007; Barton et al., 2008; McTaggart-Cowan et al., 2008; Polley et al., 2008; Heyworth et al., 2009; Ferreira et al., 2010; Chen et al., 2011; van der Meer et al., 2011; Allegra et al., 2012; Bime et al., 2012; Gonzalez-Barcala et al., 2012; Al-kalemji et al., 2013; Doz et al., 2013; Sullivan et al., 2013; D'Amato et al., 2014; Koskela et al., 2014; Peters et al., 2014; Sadatsafavi et al., 2015; Sullivan et al., 2016; Yong and Shafie, 2016; Kaambwa et al., 2017; Mitchell et al., 2017; Thomas et al., 2017; Chen et al., 2018; Chung and Han, 2018; Gray et al., 2018; Hernandez et al., 2018; Khan and Richardson, 2018; Kontodimopoulos et al., 2018; Mungan et al., 2018; Retzler et al., 2018; Tarraf et al., 2018; Wilson et al., 2018; Hernandez et al., 2019; Johnson et al., 2019; Lanario et al., 2020; Lucas et al., 2020). Of these, 40 studies capable of quantitative synthesis were meta-analyzed after excluding 12 studies without information on the measure of uncertainty. The main characteristics of the 52 included studies are presented in Table 1 (see Supplementary Tables S4 for study-level characteristics, including the study design and patient characteristics). Studies were actively conducted in Europe (44.2%) and most of them were observational designs (88.5%). EQ-5D-3L was the most used instrument (24.5%) when comparing tools that measure utility indirectly, followed by HUI-3, SF-6D, EQ-5D-5L, and ASUI. Among the direct instruments, the most frequently applied was EQ-VAS (20.8%). Numerous studies had adequate reporting for the quality assessment criteria, however, they frequently lacked an explanation for how they handled missing values (Supplementary Tables S5).

**TABLE 1:** Study characteristics (N = 52).

	Number of studies	%
Study design
Observational	46	88.5
Experimental	6	11.5
Study location		
Europe	23	44.2
North America	17	32.7
Asia	2	3.8
Multi-country	10	19.2
Publication year		
2011–2020	31	59.6
2001–2010	17	32.7
≤2000	4	7.7
Utility instrument^a^		
Indirect measures		
EQ-5D-3L	26	24.5
EQ-5D-5L	7	6.6
HUI-3	8	7.5
HUI-2	1	0.9
SF-6D	8	7.5
ASUI	6	5.7
AQL-5D	5	4.7
15D	4	3.8
QWB	1	0.9
Direct measures		
EQ-VAS	22	20.8
VAS	8	7.5
SG	6	5.7
TTO	4	3.8

*ASUI*, asthma symptom utility index; *AQL-5D*, Asthma Quality of Life Utility Index 5 Dimensions, *EQ-5D-3L* EQ-5D-3-level version, *EQ-5D-5L* EQ-5D-5-level version, *HUI*, health utilities index; *QWB*, Quality of Well-Being; *SF-6D*, Short Form-6D, *SG*, standard gamble; *TTO*, Time Trade-Off; *VAS*, visual analog scale, *15D* 15 dimensional.

^a^ Multiple studies have reported utility values using more than one instrument.

### 3.2 Meta-analyses of health state utility values

**FIGURE 2:**
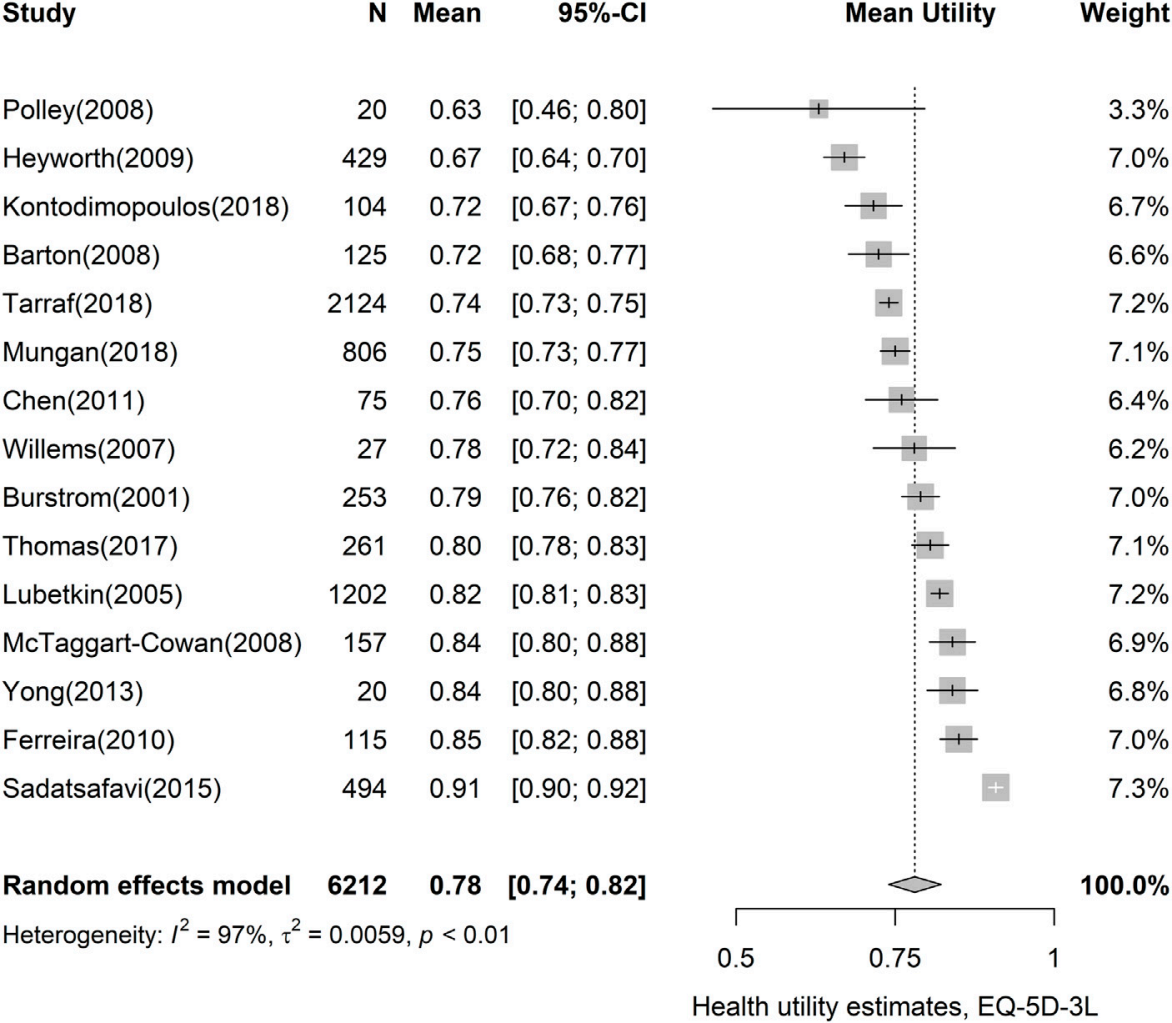
Forest plot of asthma utility, using the EQ-5D-3L instrument. CI, confidence interval; EQ-5D-3L, EQ-5D-3-level version.

Of the 40 studies included in this meta-analysis, 67 utility estimates representing general asthma were identified. Table 2 shows the results of the random effects meta-analyses using utility instruments with 95% CIs and the ranges of observed utility estimates in the studies. The most widely used instrument EQ-5D-3L resulted in a utility value of 0.78 (95% CI, 0.74–0.82). EQ-5D-5L, which has been available since 2011, showed a narrower CI than the original EQ-5D value set (95% CI, 0.83–0.86). Other measures commonly used in economic evaluations, such as HUI-3 (pooled utility, 0.78; 95% CI, 0.71–0.86) and SF-6D (pooled utility, 0.74; 95% CI, 0.70–0.79), revealed similar pooled utilities. The analysis of EQ-5D-3L showed considerable heterogeneity (I^2^: 97.4%), as results vary from 0.63 (95% CI, 0.46–0.80) to 0.91 (95% CI, 0.90–0.92) in Figure 2. Forest plots of the other utility measures are presented in Supplementary Figure S1.1–S1.10.

**TABLE 2:** Results of random effects meta-analyses for asthma utility^a^ stratified by utility instruments.

	Number of studies^b^	Number of respondents	Pooled utility, mean (95%CI)	Observed utility	
				Minimum	Maximum
Indirect measures
EQ-5D-3L	15	6,212	0.78 (0.74–0.82)	0.63	0.91
EQ-5D-5L	5	2,788	0.84 (0.83–0.86)	0.83	0.88
HUI-3	8	5,106	0.78 (0.71–0.86)	0.57	0.96
HUI-2	1	161	0.84 (0.81–0.87)	0.84	0.84
SF-6D	7	1,963	0.74 (0.70–0.79)	0.69	0.86
ASUI	5	3,089	0.76 (0.73–0.80)	0.63	0.83
AQL-5D	3	755	0.88 (0.83–0.93)	0.85	0.92
15D	2	1,435	0.84 (0.83–0.86)	0.84	0.85
QWB	1	579	0.63 (0.62–0.64)	0.63	0.63
Direct measures					
EQ-VAS	12	5,536	0.70 (0.68–0.73)	0.60	0.77
SG	4	227	0.84 (0.77–0.91)	0.49	0.91
VAS	2	176	0.60 (0.31–0.88)	0.44	0.73
TTO	2	169	0.86 (0.76–0.96)	0.81	0.91

*ASUI*, asthma symptom utility index; *AQL-5D*, Asthma Quality of Life Utility Index 5 Dimensions; *CI*, confidence interval, *EQ-5D-3L* EQ-5D-3-level version, *EQ-5D-5L* EQ-5D-5-level version, *HUI*, health utilities index; *QWB*, Quality of Well-Being; *SF-6D*, Short Form-6D, *SG*, standard gamble; *TTO*, Time Trade-Off; *VAS*, visual analog scale, *15D* 15 dimensional.

^a^ Utility of health conditions representing general asthma.

^b^ Multiple studies have reported utility values using more than one instrument.

**FIGURE 3:**
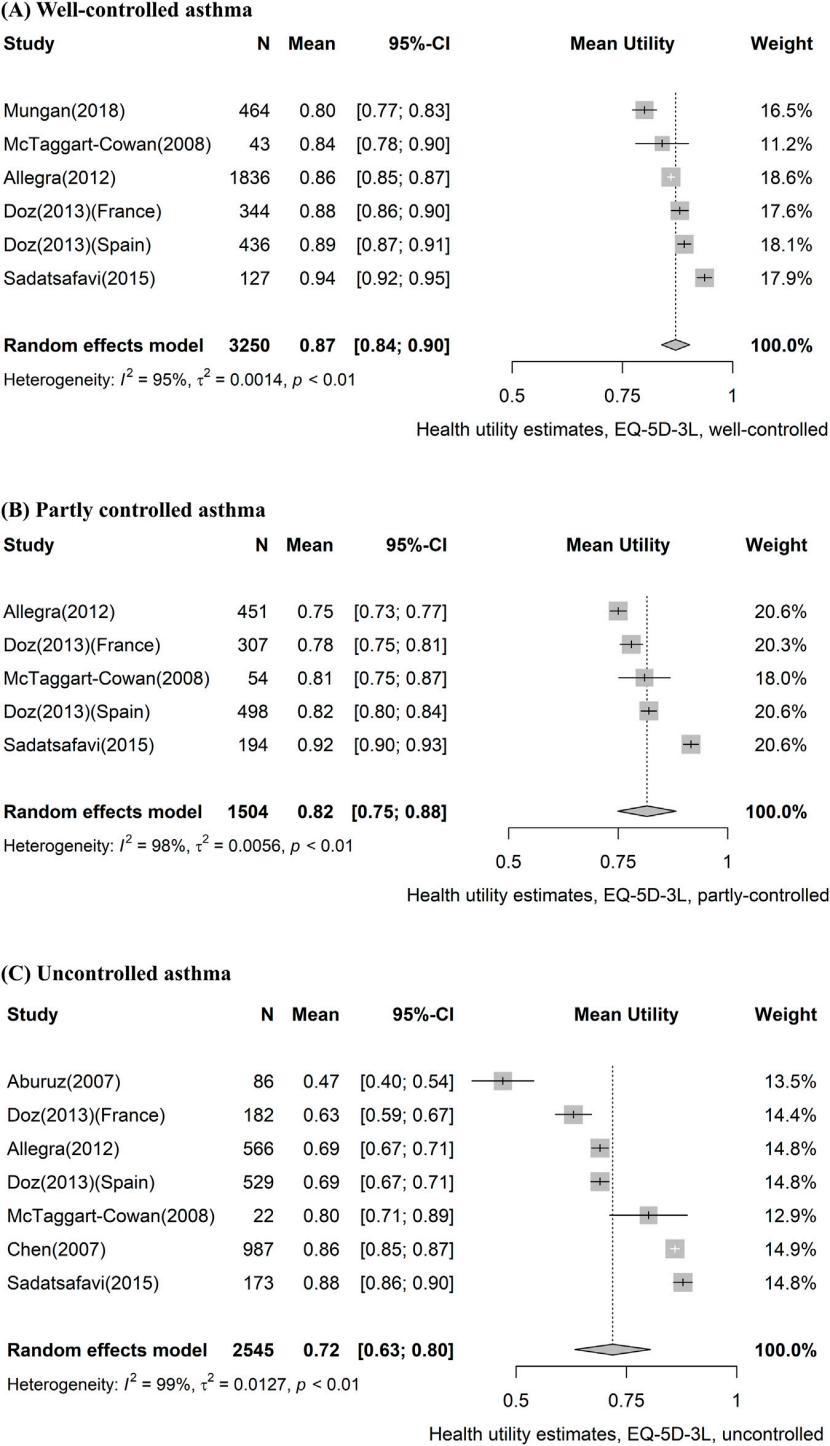
Forest plot of asthma utility stratified by control level, using the EQ-5D-3L instrument. **(A)** Well-controlled asthma, **(B)** partly controlled asthma, and **(C)** uncontrolled asthma. CI, confidence interval; EQ-5D-3L, EQ-5D-3-level version.

Table 3 shows the pooled utility values according to asthma severity and level of control. Eleven studies reported utility values according to asthma control, of which seven used EQ-5D-3L. We assessed that the more difficult it was to control asthma, the lower the pooled utility value. The most reported EQ-5D-3L values declined in the order of 0.87 (95% CI, 0.84–0.90) for well-controlled, 0.82 (95% CI, 0.75–0.88) for partly controlled, and 0.72 (95% CI, 0.63–0.80) for uncontrolled asthma. Forest plots of EQ-5D-3L values classified by control level are presented in Figure 3. Additionally, the results of the meta-analyses for two or more studies using the same measures for specific health states are provided in Supplementary Figure S2.1–S2.11.

**TABLE 3:** Results of random effects meta-analyses stratified by asthma severity and control level^a^.

	EQ-5D-3L		EQ-5D-5L		SF-6D		HUI-3		ASUI		AQL-5D	
	No. of studies	Pooled utility, mean (95% CI)	No. of studies	Pooled utility, mean (95% CI)	No. of studies	Pooled utility, mean (95% CI)	No. of studies	Pooled utility, mean (95% CI)	No. of studies	Pooled utility, mean (95% CI)	No. of studies	Pooled utility, mean (95% CI)
Severity category
Intermittent	-	-	-	-	-	-	-	-	1	0.85 (0.83–0.87)	-	-
Mild	1	0.89 (0.84–0.94)	-	-	1	0.80 (0.78–0.82)	2	0.75 (0.49–1.00)	2	0.75 (0.58–0.92)	1	0.87 (0.85–0.89)
Moderate	1	0.81 (0.75–0.87)	-	-	1	0.78 (0.76–0.80)	2	0.72 (0.46–0.97)	2	0.75 (0.65–0.86)	1	0.83 (0.81–0.85)
Severe	2	0.64 (0.42–0.86)	1	0.68 (0.63–0.73)	1	0.75 (0.70–0.80)	2	0.62 (0.37–0.88)	2	0.60 (0.39–0.82)	1	0.74 (0.67–0.81)
Control level category												
Well-controlled	5	0.87 (0.84–0.90)	1	0.93 (0.91–0.95)	1	0.79 (0.76–0.82)	1	0.83 (0.77–0.89)	-	-	2	0.93 (0.84–1.01)
Partly controlled	4	0.82 (0.75–0.88)	1	0.87 (0.85–0.89)	1	0.78 (0.76–0.80)	1	0.84 (0.80–0.88)	-	-	2	0.87 (0.75–0.99)
Uncontrolled^b^	6	0.72 (0.63–0.80)	2	0.69 (0.50–0.87)	1	0.77 (0.73–0.81)	1	0.84 (0.77–0.91)	-	-	2	0.83 (0.74–0.93)

*ASUI*, asthma symptom utility index; *AQL-5D*, Asthma Quality of Life Utility Index 5 Dimensions; *CI*, confidence interval, *EQ-5D-3L* EQ-5D-3-level version, *EQ-5D-5L* EQ-5D-5-level version, *HUI*, health utilities index, *No.* number, *SF-6D*, Short Form-6D.

^a^ Meta-analyses were performed when more than two articles were included.

^b^ Studies reporting utility values for difficult asthma were analyzed considering uncontrolled asthma. (Aburuz et al., 2007: difficult asthma; Chen et al., 2007: severe or difficult-to-treat asthma).

### 3.3 Publication bias and sensitivity analysis

The majority of valuation instruments had few reported utility values to conduct a publication bias assessment. Funnel plots and Egger’s regression test for funnel plot asymmetry did not show substantial asymmetry (Supplementary Figure S3.1, S3.2).

We excluded studies that did not explicitly report the control-level criteria for the meta-analyses as a sensitivity analysis. One study was excluded for well-controlled and partly controlled asthma and two studies were excluded for uncontrolled asthma. In this sensitivity analysis, the pooled estimates were similar, as the main analysis and the value of uncontrolled asthma only increased slightly by 0.01–0.03 (Supplementary Figure S4.1–S4.3). We also applied the leave-one-out method for sensitivity analysis of meta-analyses of EQ-5D-3L stratified by the level of asthma control. This revealed that Sadatsafavi et al. (Sadatsafavi et al., 2015) has substantial heterogeneity in the meta-analysis for well-controlled and partly controlled health states. The meta-analyses excluding the study resulted in reduced heterogeneity in both well-controlled (pooled utility, 0.86; I^2^, 89.2%) and partly controlled (pooled utility, 0.79; I^2^, 90.0%) health states (Supplementary Figure S5.1–S5.3).

## 4 Discussion

In this study, we conducted a comprehensive review of the available data on the utility values of adult asthma patients. We also performed meta-analyses according to utility instruments and health states based on the level of asthma control and severity, including various utility instruments. Many studies reporting utility values are not big enough to provide convincing estimates for each utility instrument. We provided more accurate estimates of the mean utility values and the associated uncertainty than individual studies by pooling relatively homogeneous utility values. In economic evaluations, it is recommended to use utility values obtained from studies using the same utility instrument and weights for all health states (Brazier et al., 2019). However, it may not always be possible. Our results of meta-analyses for each utility instrument could be applied to economic evaluations cautiously when appropriate utility values from the same measure are unavailable. Also, our pooled estimates and catalog of studies reporting preference-based utility values would provide a reference to determine utility values or to use instruments.

Our findings highlight the differences in utility values across different severity and levels of asthma control. Previous studies have shown that the quality of life in asthma patients decreases with decreasing levels of control and increasing severity (Juniper et al., 1993; Moy et al., 2004; Chen et al., 2007; Chen et al., 2015). Consistent with previous literature, the meta-analyses results of EQ-5D-3L, EQ-5D-5L, and SF-6D showed that utilities declined with worsened control level and severity in asthma patients. The results of meta-analyses using disease-specific instruments (ASUI, AQL-5D) also showed that utilities declined with worsened control level and severity, as with other instruments. However, only a small number of studies were included in the analysis. In the case of HUI-3, the utility of the partly controlled category (pooled utility, 0.84; 95% CI, 0.80–0.88) was marginally higher than that of the well-controlled category (pooled utility, 0.83; 95% CI, 0.77–0.89). However, these results are based on one study (McTaggart-Cowan et al., 2008), and it was reported that the difference by control level was not statistically significant.

Certain studies reported utility values that differed considerably from the EQ-5D-3L pooled estimates. For example, Sadatsafavi et al. (Sadatsafavi et al., 2015) reported a utility value of 0.91 (95% CI, 0.90–0.92) for asthma patients, which is relatively higher than the pooled estimate of 0.78 (95% CI, 0.74–0.82). It was a prospective observational study reporting 12 months of follow-up. Therefore, there is a risk of healthy volunteer bias, as patients who are able to visit the study site would be primarily included in the study (Pinsky et al., 2007). This may cause heterogeneity when compared with the results of survey-based research. In contrast, Polley et al. (2008) showed a relatively low utility value with a large standard deviation, reflecting the low precision of the estimate (pooled utility, 0.63; 95% CI, 0.46–0.80). This large variance could be due to the small sample size, i.e., 20. According to previous studies, sample size is one of the main criteria for quality assessment and is generally judged based on whether the sample size is 100 or more (Papaioannou et al., 2013; Meregaglia and Cairns, 2017; Szabo et al., 2020).

The use of various utility instruments in an economic evaluation can cause spurious results because differences between utility instruments can affect the results (Brazier et al., 2019). Therefore, it is necessary to select an appropriate utility instrument. Our study shows that EQ-5D-3L and EQ-5D-5L are appropriate for economic evaluations in terms of availability and variability of information, respectively. Economic evaluations often face difficulties in collecting optimal health state utility values, and it is difficult for a single source to reflect all the data required for decision making (Sculpher et al., 2006; Petrou et al., 2018; Brazier et al., 2019). Therefore, it is crucial to use a utility instrument with more available input values. According to this review, the most commonly used instrument in the literature reporting utility values stratified by the level of asthma control was the EQ-5D-3L; it is relevant, as utility values according to the level of asthma control are required in several economic evaluations of asthma (Gerzeli et al., 2012; Ismaila et al., 2014; Willson et al., 2014). EQ-5D-5L also appears to have advantages for use in economic evaluations. Considering that the EQ-5D-5L has been used since 2011 (Herdman et al., 2011), it has also been reported in several studies. Moreover, the 95% CI of pooled utility using EQ-5D-5L (0.83–0.86) was narrower than that using EQ-5D-3L (0.74–0.82). This was the narrowest 95% CI, except for QWB, which was reported in only one study. Therefore, when EQ-5D-5L is used for economic evaluation, it will show less uncertainty.

There were certain limitations of this review. First, high heterogeneity was observed in the meta-analyses although we used various approaches to address heterogeneity. We used strict inclusion criteria extracting the first measurement or baseline utility to use comparable utilities not confounded by further treatment. Also, meta-analyses were performed with the same utility instruments in similar disease states stratified by control level and severity. Random-effects meta-analysis were used to incorporate heterogeneity among studies that cannot be explained. Sensitivity analyses were conducted to explore heterogeneity. However, caution should be exercised when interpreting the results of the meta-analyses. The heterogeneity among the studies may be due to differences in tariffs in different countries. Furthermore, since the meta-analyses included studies regardless of severity and control-level criteria, it may cause some heterogeneity. The result of the sensitivity analysis was robust when we excluded studies that did not explicitly report the control-level criteria, but this may be due to the small number of studies excluded. Second, there may be bias in the results owing to the lack of information on standard deviations that were excluded in the meta-analyses. However, we attempted to minimize bias by calculating the standard deviation using the CI or standard error. Third, there is an assumption under the meta-analyses that continuous outcomes have a normal distribution. In meta-analyses with a small number of studies, it was difficult to prove that the assumption of normality was met. Finally, there is a risk of publication bias as an inherent limitation of the meta-analyses. However, the results of Egger’s test showed that there was no substantial small study effect.

## 5 Conclusion

This systematic review provides a comprehensive overview of the utility values in asthma. Among utility instruments, EQ-5D-3L had an advantage in terms of information availability, and EQ-5D-5L was expected to show less uncertainty. Utility values declined with worsened control level or in more severe asthma patients. This study will provide a useful resource for health economists conducting economic evaluations of asthma treatments.

## Data Availability

The original contributions presented in the study are included in the article/Supplementary Material, further inquiries can be directed to the corresponding authors.
