# Supplementary material for: Health-related quality of life in adult patients with asthma according to asthma control and severity: A systematic review and meta-analysis
Source: Front Pharmacol. 2022 Nov 21;13:908837. doi: 10.3389/fphar.2022.908837 (PMC9720394; doi:10.3389/fphar.2022.908837)
Supplement: Supplementary file 1 [file Table1.DOCX]

Supplementary Material

Supplementary Table 1: Eligibility criteria

Supplementary Table 2: Search strategies

Supplementary Table 3: Citations of excluded full texts

Supplementary Table 4: Study characteristics

Supplementary Table 5: Results of quality assessment

Supplementary Figure 1: Forest plots of asthma utility representing asthma

Supplementary Figure 2: Forest plots for each utility instrument stratified by severity and control level

Supplementary Figure 3: Funnel plots

Supplementary Figure 4: Sensitivity analysis excluding studies with unspecified control level criteria

Supplementary Figure 5: Sensitivity analysis using the leave-one-out method

***Supplementary Table 1: Eligibility criteria***

**Supplementary Table S1. Inclusion and exclusion criteria**

| **Characteristic** | **Inclusion criteria** | **Exclusion criteria** |
| --- | --- | --- |
| Population | Adults (≥18 years) with asthma  General population reporting the valuation of changes in patients’ health-related quality of life  Experts reporting the valuation of changes in patients’ health-related quality of life | Adolescents, Children (<18 years) |
| Intervention/Comparator | - | - |
| Outcomes | Studies measuring at least one component of quality of life of asthma using predefined utility instruments (EQ-5D-3L, EQ-5D-5L, HUI-2, HUI-3, SF-6D, ASUI, AQL-5D, 15D, QWB, EQ-VAS, VAS, SG, TTO) either as a primary or secondary outcome.  Randomized controlled trials, non-randomized controlled trials, cohort studies, case-control studies, and cross-sectional studies. | Studies using mapping algorithm to calculate preference-based health utilities.  Review articles, case series, case reports, editorials, comments, and letters.  Conference abstracts and articles unavailable in full text. |
| Publication type | Original research  Secondary research only if citing unpublished results from original research  Only full-text publications | Publications with HSUVs reported in other included studies (duplicate citations)  Review articles, case series, case reports, editorials, comments, and letters. Conference abstracts and articles unavailable in full text. |
| Date limits | Unlimited | - |
| Language | English only | - |
| EQ-5D, European Quality of Life 5 Dimensions; VAS, Visual Analog Scale; HUI, Health Utilities Index; SF-6D, Short Form-6D; ASUI, Asthma Symptom Utility Index; SG, Standard Gamble; AQL-5D, Asthma Quality of Life Utility Index 5 Dimensions; 15D, 15 dimensional; TTO, Time Trade-Off; QWB, Quality of Well-Being; HSUVs, Health-state Utility Values | | |

***Supplementary Table 2: Search strategies***

**Supplementary Table S2.1. PubMed search strategy**

| Date of search: July, 07, 2020 | | |
| --- | --- | --- |
| PubMed (Medline) | | |
| # | Search | Result |
| #1 | "Asthma"[MeSH] | 127,636 |
| #2 | Asthma[TW] OR Asthmas[TW] OR Asthmatic[TW] | 178,240 |
| #3 | Search (#1 OR #2) | 178,490 |
| #4 | “Quality of Life”[MeSH] | 194,086 |
| #5 | “Quality of Life”[TW] | 330,196 |
| #6 | “Life Quality”[TW] OR HRQOL[TW] OR “Health-Related Quality Of Life”[TW] OR “Health Related Quality Of Life”[TW] OR QOL[TW] OR HQL[TW] OR HQOL[TW] OR HRQL[TW] | 84,718 |
| #7 | Search (#4 OR #5 OR #6) | 334,646 |
| #8 | utilit*[TW] | 207,456 |
| #9 | Search (#7 OR #8) | 533,077 |
| #10 | “time-trade-off”[TW] OR “time trade off”[TW] OR “time trade-off”[TW] OR “time tradeoff”[TW] OR TTO[TW] | 1,887 |
| #11 | “standard gamble”[TW] OR standard-gamble[TW] | 849 |
| #12 | HUI2[TW] OR HUI3[TW] OR “HUI 2”[TW] OR “HUI 3”[TW] OR “HUI II”[TW] OR “HUI III”[TW] OR “HUI-2”[TW] OR “HUI-3”[TW] OR “HUI-II”[TW] OR “HUI-III”[TW] OR “HUI Mark 2”[TW] OR “HUI Mark 3”[TW] OR “Health Utilities Index”[TW] OR “Health Utility Index”[TW] | 989 |
| #13 | SF-6D*[TW] OR SF6D*[TW] OR “SF 6D”[TW] OR “short form 6D”[TW] OR “shortform 6D”[TW] OR “SF six D”[TW] OR SFsixD[TW] OR “shortform six D”[TW] OR “short form six D”[TW] | 862 |
| #14 | “EQ 5D”[TW] OR “EQ-5D”[TW] OR EQ5D[TW] OR “EQol 5D”[TW] OR “European Quality of Life”[TW] OR “EuroQol”[TW] OR “Euro Qol”[TW] OR “Euro-Qol”[TW] | 11,309 |
| #15 | “visual analogue scale”[TW] OR “visual analog scale”[TW] OR “visual analog scaling”[TW] OR VAS[TW] | 74,697 |
| #16 | “Quality of Well-Being”[TW] OR “Quality of Wellbeing”[TW] OR “Quality of Well Being”[TW] OR QWB[TW] | 382 |
| #17 | 15-D[TW] OR 15D[TW] | 4,914 |
| #18 | ASUI[TW] OR “Asthma Symptom Utility”[TW] | 25 |
| #19 | “AQL-5D”[TW] OR “AQL 5D”[TW] AQL5D[TW] OR “Asthma Quality of Life Utility Index”[TW] | 14 |
| #20 | Search (#10 OR #11 OR #12 OR #13 OR #14 OR #15 OR #16 OR #17 OR #18 OR #19) | 90,661 |
| #21 | Search(#9 AND #20) | 19,888 |
| #22 | Search(#3 AND #21) | 302 |
| #23 | Search(#3 AND #21) Filters: English | 286 |

**Supplementary Table S2.2. Embase search strategy**

| Date of search: July, 07, 2020 | | |
| --- | --- | --- |
| Embase | | |
| # | Search | Result |
| #1 | ‘asthma’/exp OR ‘asthma’ OR ‘asthmas’ OR ‘asthmatic’/exp OR ‘asthmatic’ | 327,667 |
| #2 | ‘quality of life’/exp OR ‘quality of life’ OR ‘life quality’ OR hrqol OR ‘health-related quality of life’ OR ‘health related quality of life’ OR qol OR hql OR hqol OR hrql | 605,107 |
| #3 | utilit* | 295,267 |
| #4 | #2 OR #3 | 877,488 |
| #5 | ‘time-trade-off’ OR ‘time trade off’ OR ‘time trade-off’ OR ‘time tradeoff’ OR tto | 2,828 |
| #6 | ‘standard gamble’ OR ‘standard-gamble’ | 1,116 |
| #7 | hui2 OR hui3 OR ‘hui 2’ OR ‘hui 3’ OR ‘hui II’ OR ‘hui III’ OR ‘hui-2’ OR ‘hui-3’ OR ‘hui-II’ OR ‘hui-III’ OR ‘hui mark 2’ OR ‘hui mark 3’ OR ‘health utilities index’ OR ‘health utility index’ | 1,459 |
| #8 | ‘sf 6d*’ OR sf6d* OR ‘sf 6d’ OR ‘short form 6d’ OR ‘shortform 6d’ OR ‘sf six d’ OR sfsixd OR ‘shortform six d’ OR ‘short form six d’ | 1,579 |
| #9 | ‘european quality of life 5 dimensions questionnaire’/exp OR ‘eq 5d’ OR ‘eq-5d’ OR eq5d OR ‘eqol 5d’ OR ‘european quality of life’ OR euroqol OR ‘euro qol’ OR euro-qol | 21,829 |
| #10 | ‘visual analogue scale’ OR ‘visual analog scale’ OR ‘visual analog scaling’ OR vas | 144,020 |
| #11 | ‘quality of well-being’ OR ‘quality of wellbeing’ OR ‘quality of well being’ OR qwb | 482 |
| #12 | ‘15-d’ OR 15d | 7,455 |
| #13 | asui OR 'asthma symptom utility' | 119 |
| #14 | ‘aql-5d’ OR ‘aql 5d’ OR aql5d OR ‘asthma quality of life utility index’ | 16 |
| #15 | #5 OR #6 OR #7 OR #8 OR #9 OR #10 OR #11 OR #12 OR #13 OR #14 | 170,643 |
| #16 | #4 AND #15 | 41,661 |
| #17 | #1 AND #16 | 870 |
| #18 | #1 AND #16 AND [english]/lim | 846 |

**Supplementary Table S2.3. CENTRAL search strategy**

| Date of search: July, 07, 2020 | | |
| --- | --- | --- |
| Cochrane (CENTRAL) | | |
| # | Search | Result |
| #1 | MeSH descriptor: [Asthma] explode all trees | 11,603 |
| #2 | Asthma OR Asthmas OR Asthmatic | 35,324 |
| #3 | #1 OR #2 | 35,329 |
| #4 | MeSH descriptor: [Quality of Life] explode all trees | 23,373 |
| #5 | “Quality of Life” OR “Life Quality” OR HRQOL OR “Health-Related Quality Of Life” OR “Health Related Quality Of Life” OR QOL OR HQL OR HQOL OR HRQL | 113,898 |
| #6 | #4 OR #5 | 113,898 |
| #7 | utilit* | 15,209 |
| #8 | #6 OR #7 | 125,284 |
| #9 | “time-trade-off” OR “time trade off” OR “time trade-off” OR “time tradeoff” OR TTO | 278 |
| #10 | “standard gamble” OR standard-gamble | 92 |
| #11 | HUI2 OR HUI3 OR “HUI 2” OR “HUI 3” OR “HUI II” OR “HUI III” OR “HUI-2” OR “HUI-3” OR “HUI-II” OR “HUI-III” OR “HUI Mark 2” OR “HUI Mark 3” OR “Health Utilities Index” OR “Health Utility Index” | 322 |
| #12 | SF-6D* OR SF6D* OR “SF 6D” OR “short form 6D” OR “shortform 6D” OR “SF six D” OR SFsixD OR “shortform six D” OR “short form six D” | 325 |
| #13 | “EQ 5D” OR “EQ-5D” OR EQ5D OR “EQol 5D” OR “European Quality of Life” OR “EuroQol” OR “Euro Qol” OR “Euro-Qol” | 9,522 |
| #14 | “visual analogue scale” OR “visual analog scale” OR “visual analog scaling” OR VAS | 55,937 |
| #15 | “Quality of Well-Being” OR “Quality of Wellbeing” OR “Quality of Well Being” OR QWB | 137 |
| #16 | “15-D” OR 15D | 5,280 |
| #17 | ASUI OR “Asthma Symptom Utility” | 27 |
| #18 | “AQL-5D” OR “AQL 5D” OR AQL5D OR “Asthma Quality of Life Utility Index” | 0 |
| #19 | #9 OR #10 OR #11 OR #12 OR #13 OR #14 OR #15 OR #16 OR #17 | 67,752 |
| #20 | #8 AND #18 | 18,530 |
| #21 | #3 AND #19 | 666 |
| #22 | #3 AND #19  in Trials | 238 |

***Supplementary Table 3: Citations of excluded full texts***

**Supplementary Table S3. Citations of excluded full texts with reasons for exclusion**

| **#** | **Citation** | **Reason for exclusion** |
| --- | --- | --- |
| 1 | Ainsworth B, Greenwell K, Stuart B, Raftery J, Mair F, Bruton A, Yardley L, Thomas M. Feasibility trial of a digital self-management intervention 'My Breathing Matters' to improve asthma-related quality of life for UK primary care patients with asthma. BMJ Open. 2019 Nov 12;9(11):e032465. | Clinically or methodologically incomparable to other studies |
| 2 | Almomani BA, Al-Sawalha NA, Samrah SM, Gamble JM, Al Momani MA. Asthma insights from Jordan: cross-sectional observational study. J Asthma. 2016;53(4):349-55. | Data not applicable |
| 3 | Bjermer L, Lemiere C, Maspero J, Weiss S, Zangrilli J, Germinaro M. Reslizumab for Inadequately Controlled Asthma With Elevated Blood Eosinophil Levels: A Randomized Phase 3 Study. Chest. 2016 Oct;150(4):789-798. | Clinically or methodologically incomparable to other studies |
| 4 | Brazier J, Rowen D, Yang Y, Tsuchiya A. Comparison of health state utility values derived using time trade-off, rank and discrete choice data anchored on the full health-dead scale. Eur J Health Econ. 2012;13(5):575-587. | No utility data available |
| 5 | Brusselle G, Michils A, Louis R, Dupont L, Van de Maele B, Delobbe A, Pilette C, Lee CS, Gurdain S, Vancayzeele S, Lecomte P, Hermans C, MacDonald K, Song M, Abraham I. "Real-life" effectiveness of omalizumab in patients with severe persistent allergic asthma: The PERSIST study. Respir Med. 2009 Nov;103(11):1633-42. | Clinically or methodologically incomparable to other studies |
| 6 | Burn J, Sims AJ, Patrick H, Heaney LG, Niven RM. Efficacy and safety of bronchial thermoplasty in clinical practice: a prospective, longitudinal, cohort study using evidence from the UK Severe Asthma Registry. BMJ Open. 2019 Jun 19;9(6):e026742. | Clinically or methodologically incomparable to other studies |
| 7 | Canonica GW, Bartezaghi M, Marino R, Rigoni L. Prevalence of perennial severe allergic asthma in Italy and effectiveness of omalizumab in its management: PROXIMA - an observational, 2 phase, patient reported outcomes study. Clin Mol Allergy. 2015;13(1):10. Published 2015 Jul 7. | No utility data available |
| 8 | Chen W, Lynd LD, FitzGerald JM, Marra CA, Rousseau R, Sadatsafavi M. The added effect of comorbidity on health-related quality of life in patients with asthma. Qual Life Res. 2015 Oct;24(10):2507-17. | Not original data |
| 9 | Colombo D, Zagni E, Ferri F, Canonica GW. Gender differences in asthma perception and its impact on quality of life: a post hoc analysis of the PROXIMA (Patient Reported Outcomes and Xolair(®) In the Management of Asthma) study. Allergy Asthma Clin Immunol. 2019;15:65. | Clinically or methodologically incomparable to other studies |
| 10 | Crossman-Barnes CJ, Sach T, Wilson A, Barton G. The construct validity and responsiveness of the EQ-5D-5L, AQL-5D and a bespoke TTO in acute asthmatics. Qual Life Res. 2020 Mar;29(3):619-627. Epub 2019 Nov 2. | Clinically or methodologically incomparable to other studies |
| 11 | DiBonaventura M, Luo X, Moffatt M, Bushmakin AG, Kumar M, Bobula J. The Association Between Vulvovaginal Atrophy Symptoms and Quality of Life Among Postmenopausal Women in the United States and Western Europe. J Womens Health (Larchmt). 2015;24(9):713-722. | No utility data available |
| 12 | Efthymiadou, Olina & Mossman, Jean & Kanavos, Panos. Health related quality of life aspects not captured by EQ-5D-5L: Results from an international survey of patients. Health Policy. 2018 | No utility data available |
| 13 | El Rhazi K, Nejjari C, Benjelloun MC, et al. Validation of the St. George's Respiratory Questionnaire in patients with COPD or asthma in Morocco. Int J Tuberc Lung Dis. 2006;10(11):1273-1278. | Not asthma |
| 14 | Faria R, McKenna C, Palmer S. Optimizing the position and use of omalizumab for severe persistent allergic asthma using cost-effectiveness analysis. Value Health. 2014 Dec;17(8):772-82. Epub 2014 Oct 11. | Not original data |
| 15 | Ferreira LN, Ferreira PL, Pereira LN. Comparing the performance of the SF-6D and the EQ-5D in different patient groups. Acta Med Port. 2014 Mar-Apr;27(2):236-45. Epub 2014 Apr 30. | Not original data |
| 16 | Ferreira PL, Ferreira LN, Pereira LN. Contributos para a Validação da Versão Portuguesa do EQ-5D [Contribution for the validation of the Portuguese version of EQ-5D]. Acta Med Port. 2013;26(6):664-675. | No utility data available |
| 17 | Flanagan W, McIntosh CN, Le Petit C, Berthelot JM. Deriving utility scores for co-morbid conditions: a test of the multiplicative model for combining individual condition scores. Popul Health Metr. 2006 Oct 31;4:13. | Insufficient data to analyze |
| 18 | Garratt AM, Hutchinson A, Russell I. Patient-assessed measures of health outcome in asthma: a comparison of four approaches. Respir Med. 2000 Jun;94(6):597-606. | Clinically or methodologically incomparable to other studies |
| 19 | Gelincik A, Demir S, Olgaç M, et al. High adherence to subcutaneous immunotherapy in a real-life study from a large tertiary medical center. Allergy Asthma Proc. 2017;38(6):78-84. | Comorbidities |
| 20 | Guertin JR, Humphries B, Feeny D, Tarride JÉ. Health Utilities Index Mark 3 scores for major chronic conditions: Population norms for Canada based on the 2013 and 2014 Canadian Community Health Survey. Health Rep. 2018 Nov 21;29(11):12-19. | No sample size available |
| 21 | Hays, RD, Reeve, BB, Smith, AW, et al. Associations of cancer and other chronic medical conditions with SF-6D preference-based scores in Medicare beneficiaries. Qual Life Res 23, 385–391 (2014). | No utility data available |
| 22 | Hernández Alava M, Wailoo A, Pudney S, Gray L, Manca A. Mapping clinical outcomes to generic preference-based outcome measures: development and comparison of methods. Health Technol Assess. 2020;24(34):1-68. | No utility data available |
| 23 | Hirai K, Shirai T, Suzuki Y, Shimomura T, Itoh K. Comparison of the Association between Circulating Vitamin D3 Levels and Clinical Outcomes in Patients with Asthma and Chronic Obstructive Pulmonary Disease: A Prospective Observational Study. Biol Pharm Bull. 2019;42(11):1861-1866. | No utility data available |
| 24 | Hyland ME, Jones RC, Lanario JW, Masoli M. The construction and validation of the Severe Asthma Questionnaire. Eur Respir J. 2018 Jul 11;52(1):1800618. | Clinically or methodologically incomparable to other studies |
| 25 | Ilmarinen P, Juboori H, Tuomisto LE, Niemelä O, Sintonen H, Kankaanranta H. Effect of asthma control on general health-related quality of life in patients diagnosed with adult-onset asthma. Sci Rep. 2019 Nov 6;9(1):16107. | Clinically or methodologically incomparable to other studies |
| 26 | Ismaila AS, Risebrough N, Li C, Corriveau D, Hawkins N, FitzGerald JM, Su Z. COST-effectiveness of salmeterol/fluticasone propionate combination (Advair(®)) in uncontrolled asthma in Canada. Respir Med. 2014 Sep;108(9):1292-302. Epub 2014 Jun 25. | Not original data |
| 27 | Johnson JB, Summer W, Cutler RG, Martin B, Hyun DH, Dixit VD, Pearson M, Nassar M, Telljohann R, Maudsley S, Carlson O, John S, Laub DR, Mattson MP. Alternate day calorie restriction improves clinical findings and reduces markers of oxidative stress and inflammation in overweight adults with moderate asthma. Free Radic Biol Med. 2007 Mar 1;42(5):665-74. | Comorbidities |
| 28 | Kardos P, Wittchen HU, Mühlig S, Ritz T, Buhl R, Rabe K, Klotsche J, Riedel O; sap-NEEDS study group. Controlled and uncontrolled allergic asthma in routine respiratory specialist care - a clinical-epidemiological study in Germany. Curr Med Res Opin. 2011 Sep;27(9):1835-47. | Clinically or methodologically incomparable to other studies |
| 29 | Kauppinen R, Sintonen H, Tukiainen H. One-year economic evaluation of intensive vs conventional patient education and supervision for self-management of new asthmatic patients. Respir Med. 1998 Feb;92(2):300-7. | Clinically or methodologically incomparable to other studies |
| 30 | Kauppinen R, Vilkka V, Sintonen H, Klaukka T, Tukiainen H. Long-term economic evaluation of intensive patient education during the first treatment year in newly diagnosed adult asthma. Respir Med. 2001 Jan;95(1):56-63. | Clinically or methodologically incomparable to other studies |
| 31 | Lee LK, Ramakrishnan K, Safioti G, Ariely R, Schatz M. Asthma control is associated with economic outcomes, work productivity and health-related quality of life in patients with asthma. BMJ Open Respir Res. 2020;7(1):e000534. | Clinically or methodologically incomparable to other studies |
| 32 | Lee TA, Hollingworth W, Sullivan SD. Comparison of directly elicited preferences to preferences derived from the SF-36 in adults with asthma. Med Decis Making. 2003 Jul-Aug;23(4):323-34. | Clinically or methodologically incomparable to other studies |
| 33 | Leidy KN, Chan KS, Coughlin C. Is the asthma quality of life questionnaire a useful measure for low-income asthmatics? Am J Respir Crit Care Med. 1998 Oct;158(4):1082-90. | Clinically or methodologically incomparable to other studies |
| 34 | Leidy NK, Coughlin C. Psychometric performance of the Asthma Quality of Life Questionnaire in a US sample. Qual Life Res. 1998;7(2):127-134. | Not measured by preference-based measurement |
| 35 | Lloyd A, Doyle S, Dewilde S, Turk F. Preferences and utilities for the symptoms of moderate to severe allergic asthma. Eur J Health Econ. 2008 Aug;9(3):275-84. | Clinically or methodologically incomparable to other studies |
| 36 | Lloyd A, Price D, Brown R. The impact of asthma exacerbations on health-related quality of life in moderate to severe asthma patients in the UK. Prim Care Respir J. 2007 Feb;16(1):22-7. | Clinically or methodologically incomparable to other studies |
| 37 | Marchetti M, Cavallo M, Annoni E, Gerzeli S. Cost-utility of inhaled corticosteroids in patients with moderate-to-severe asthma. Expert Rev Pharmacoecon Outcomes Res. 2004 Oct;4(5):549-64. | No utility data available |
| 38 | McCallister JW, Holbrook JT, Wei CY, et al. Sex differences in asthma symptom profiles and control in the American Lung Association Asthma Clinical Research Centers. Respir Med. 2013;107(10):1491-1500. | Not original data |
| 39 | McColl E, Eccles MP, Rousseau NS, Steen IN, Parkin DW, Grimshaw JM. From the generic to the condition-specific?: Instrument order effects in Quality of Life Assessment. Med Care. 2003;41(7):777-790. | No utility data available |
| 40 | Mészáros A, Zelkó R, Meskó A, Vincze Z. Factorial design for the analysis of patient's quality of life in asthma. Qual Life Res. 2005;14(1):191-195. | No utility data available |
| 41 | Mitchell PM, Al-Janabi H, Richardson J, Iezzi A, Coast J. The Relative Impacts of Disease on Health Status and Capability Wellbeing: A Multi-Country Study. PLoS One. 2015;10(12):e0143590. Published 2015 Dec 2. | No utility data available |
| 42 | Mo F, Choi BC, Li FC, Merrick J. Using Health Utility Index (HUI) for measuring the impact on health-related quality of Life (HRQL) among individuals with chronic diseases. ScientificWorldJournal. 2004;4:746-757. Published 2004 Aug 27. | No utility data available |
| 43 | Mougey EB, Feng H, Castro M, Irvin CG, Lima JJ. Absorption of montelukast is transporter mediated: a common variant of OATP2B1 is associated with reduced plasma concentrations and poor response. Pharmacogenet Genomics. 2009 Feb;19(2):129-38. | Clinically or methodologically incomparable to other studies |
| 44 | Muggah E, Graves E, Bennett C, Manuel DG. Ascertainment of chronic diseases using population health data: a comparison of health administrative data and patient self-report. BMC Public Health. 2013 Jan 9;13:16. | Clinically or methodologically incomparable to other studies |
| 45 | Murphy K, Jacobs J, Bjermer L, Fahrenholz JM, Shalit Y, Garin M, Zangrilli J, Castro M. Long-term Safety and Efficacy of Reslizumab in Patients with Eosinophilic Asthma. J Allergy Clin Immunol Pract. 2017 Nov-Dec;5(6):1572-1581.e3. Erratum in: J Allergy Clin Immunol Pract. 2018 May - Jun;6(3):1095. | Clinically or methodologically incomparable to other studies |
| 46 | Niederberger V, Neubauer A, Gevaert P, et al. Safety and efficacy of immunotherapy with the recombinant B-cell epitope-based grass pollen vaccine BM32. J Allergy Clin Immunol. 2018;142(2):497-509.e9. | Comorbidities |
| 47 | Nishimura K, Oga T, Ikeda A, Hajiro T, Tsukino M, Koyama H. Comparison of health-related quality of life measurements using a single value in patients with asthma and chronic obstructive pulmonary disease. J Asthma. 2008 Sep;45(7):615-20. | No utility data available |
| 48 | Ock M, Han JW, Lee JY, Kim SH, Jo MW. Estimating quality-adjusted life-year loss due to noncommunicable diseases in Korean adults through to the year 2040. Value Health. 2015;18(1):61-66. | No utility data available |
| 49 | Oga T, Nishimura K, Tsukino M, Sato S, Hajiro T, Mishima M. A comparison of the responsiveness of different generic health status measures in patients with asthma. Qual Life Res. 2003 Aug;12(5):555-63. | Clinically or methodologically incomparable to other studies |
| 50 | Park SJ, Ahn S, Park KH. Burden of Visual Impairment and Chronic Diseases. JAMA Ophthalmol. 2016;134(7):778–784. | Not original data |
| 51 | Parkin D, Devlin N, Feng Y. What Determines the Shape of an EQ-5D Index Distribution?. Med Decis Making. 2016;36(8):941-951. | No utility data available |
| 52 | Peters M, Crocker H, Dummett S, Jenkinson C, Doll H, Fitzpatrick R. Change in health status in long-term conditions over a one year period: a cohort survey using patient-reported outcome measures. Health Qual Life Outcomes. 2014 Aug 12;12:123. | Not original data |
| 53 | Petersen KD, Kronborg C, Larsen JN, Dahl R, Gyrd-Hansen D. Patient related outcomes in a real life prospective follow up study: Allergen immunotherapy increase quality of life and reduce sick days. World Allergy Organ J. 2013;6(1):15. Published 2013 Sep 9. | Comorbidities |
| 54 | Popov TA, Petrova D, Kralimarkova TZ, Ivanov Y, Popova T, Peneva M, Odzhakova T, Ilieva Y, Yakovliev P, Lazarova T, Georgiev O, Hodzhev V, Hodzheva E, Staevska MT, Dimitrov VD. Real life clinical study design supporting the effectiveness of extra-fine inhaled beclomethasone/formoterol at the level of small airways of asthmatics. Pulm Pharmacol Ther. 2013 Dec;26(6):624-9. Epub 2013 Jun 14. | No utility data available |
| 55 | Price D, Musgrave S, Wilson E, Sims E, Shepstone L, Blyth A, Murdoch J, Mugford M, Juniper E, Ayres J, Wolfe S, Freeman D, Lipp A, Gilbert R, Harvey I. A pragmatic single-blind randomised controlled trial and economic evaluation of the use of leukotriene receptor antagonists in primary care at steps 2 and 3 of the national asthma guidelines (ELEVATE study). Health Technol Assess. 2011 May;15(21):1-132. | Clinically or methodologically incomparable to other studies |
| 56 | Quirk FH, Baveystock CM, Wilson R, Jones PW. Influence of demographic and disease related factors on the degree of distress associated with symptoms and restrictions on daily living due to asthma in six countries. Eur Respir J. 1991 Feb;4(2):167-71. | No utility data available |
| 57 | Rasulnia M, Burton BS, Ginter RP, Wang TY, Pleasants RA, Green CL, Lugogo N. Assessing the impact of a remote digital coaching engagement program on patient-reported outcomes in asthma. J Asthma. 2018 Jul;55(7):795-800. | Clinically or methodologically incomparable to other studies |
| 58 | Remenschneider AK, Scangas G, Meier JC, et al. EQ-5D-derived health utility values in patients undergoing surgery for chronic rhinosinusitis. Laryngoscope. 2015;125(5):1056-1061. | Not original data |
| 59 | Ritva K, Pekka R, Harri S. Agreement between a generic and disease-specific quality-of-life instrument: the 15D and the SGRQ in asthmatic patients. Qual Life Res. 2000;9(9):997-1003. | No utility data available |
| 60 | Rowen D, Brazier J, Tsuchiya A, Alava MH. Valuing states from multiple measures on the same visual analogue sale: A feasibility study. Health Economics. 2012;21(6):715-29. | Clinically or methodologically incomparable to other studies |
| 61 | Rowen D, Brazier J, Van Hout B. A comparison of methods for converting DCE values onto the full health-dead QALY scale. Med Decis Making. 2015;35(3):328-340. | No utility data available |
| 62 | Seston EM, Magola E, Bower P, et al. Supporting patients with long-term conditions in the community: Evaluation of the Greater Manchester Community Pharmacy Care Plan Service. Health Soc Care Community. 2020;28(5):1671-1687. | Comorbidities |
| 63 | Sommar JN, Ek A, Middelveld R, et al. Quality of life in relation to the traffic pollution indicators NO2 and NOx: results from the Swedish GA(2)LEN survey. BMJ Open Respir Res. 2014;1(1):e000039. Published 2014 Aug 14. | No utility data available |
| 64 | Sullivan PW, Kavati A, Ghushchyan VH, Lanz MJ, Ortiz B, Maselli DJ, LeCocq J. Impact of allergies on health-related quality of life in patients with asthma. J Asthma. 2020 Nov;57(11):1263-1272. | Comorbidities |
| 65 | Svedsater H, Jones R, Bosanquet N, et al. Patient-reported outcomes with initiation of fluticasone furoate/vilanterol versus continuing usual care in the Asthma Salford Lung Study. Respir Med. 2018;141:198-206. | No utility data available |
| 66 | Tsiplova K, Pullenayegum E, Cooke T, Xie F. EQ-5D-derived health utilities and minimally important differences for chronic health conditions: 2011 Commonwealth Fund Survey of Sicker Adults in Canada. Qual Life Res. 2016;25(12):3009-3016. | Comorbidities |
| 67 | Versteegh MM, Leunis A, Uyl-de Groot CA, Stolk EA. Condition-specific preference-based measures: benefit or burden? Value Health. 2012 May;15(3):504-13. | No utility data available |
| 68 | Virchow JC, McDonald M, Garin M, Korn S. Reslizumab as add-on therapy in patients with refractory asthma. BMJ Open Respir Res. 2020 Apr;7(1):e000494. | No utility data available |
| 69 | Wechsler ME, Yawn BP, Fuhlbrigge AL, Pace WD, Pencina MJ, Doros G, Kazani S, Raby BA, Lanzillotti J, Madison S, Israel E; BELT Investigators. Anticholinergic vs Long-Acting β-Agonist in Combination With Inhaled Corticosteroids in Black Adults With Asthma: The BELT Randomized Clinical Trial. JAMA. 2015 Oct 27;314(16):1720-30. | Clinically or methodologically incomparable to other studies |
| 70 | Wittchen HU, Mühlig S, Klotsche J, et al. Omalizumab versus 'usual care': results from a naturalistic longitudinal study in routine care. Int Arch Allergy Immunol. 2012;159(1):83-93. | No utility data available |
| 71 | Yang Y, Brazier JE, Tsuchiya A, Young TA. Estimating a preference-based index for a 5-dimensional health state classification for asthma derived from the asthma quality of life questionnaire. Med Decis Making. 2011 Mar-Apr;31(2):281-91. | No utility data available |
| 72 | Yazıcı ZM, Sayın I, Bozkurt E, Kayhan FT. Effect of montelukast on quality of life in subjects with nasal polyposis accompanying bronchial asthma. Kulak Burun Bogaz Ihtis Derg. 2011;21(4):210-214. | No utility data available |
| 73 | Yorke J, Adair P, Doyle AM, Dubrow-Marshall L, Fleming S, Holmes L, Menzies-Gow A, Niven R, Pilling M, Shuldham C. A randomised controlled feasibility trial of Group Cognitive Behavioural Therapy for people with severe asthma. J Asthma. 2017 Jun;54(5):543-554. Epub 2016 Nov 23. | Comorbidities |
| 74 | Young TA, Yang Y, Brazier JE, Tsuchiya A. The use of rasch analysis in reducing a large condition-specific instrument for preference valuation: the case of moving from AQLQ to AQL-5D. Med Decis Making. 2011;31(1):195-210. | No utility data available |

***Supplementary Table 4: Study characteristics***

**Supplementary Table S4. Study design and patient characteristics of included studies**

| **Author, Year** | **Country** | **Study Design** | **Mean (SD) Age, years** | **% Males** | **Respondent, N** | **Utility Instrument** | |
| --- | --- | --- | --- | --- | --- | --- | --- |
| Aburuz *et al.* ^†^  (2007) | UK | observational | 42.3 (15.0) | 38.4 | Uncontrolled: 86  (difficult asthma) | EQ-5D-3L  EQ-VAS | |
| Al-kalemji *et al.*  (2013) | Denmark | observational | n/a | 40.9 | Asthma: 181 | 15D | |
| Allegra *et al.*  (2012) | Italy | observational | 1.well-controlled: 46 (16)  2.partly controlled: 45 (15)  3.uncontrolled: 47 (15) | 1.well-controlled: 40.3  2.partly controlled: 35.5  3.uncontrolled: 32.3 | 1. well-controlled: 1836  2. partly controlled: 451  3. uncontrolled: 566 | EQ-5D-3L  EQ-VAS | |
| Barton *et al.*  (2008) | UK | observational | n/a | n/a | Asthma: 125 | EQ-5D-3L  SF-6D  EQ-VAS | |
| Bime *et al*.  (2012) | America | observational | - SIIVA trial: 42 (12)  - SARA trial: 41 (13) | - SIIVA trial: 25  - SARA trial: 32 | - SIIVA Trial(GINA)  Asthma: 1236  1. intermittent: 332  2. mild: 453  3. moderate: 298  4. severe: 137  - SARA Trial  Asthma: 412 | ASUI | |
| Blumenschein *et al.*  (1998) | UK | observational | 40.32 (15.11) | 25 | Asthma: 69 | SG  TTO | |
| Burström *et al.*  (2001) | Sweden | observational | n/a | n/a | Asthma: 253 | EQ-5D-3L | |
| Chen *et al.* ^†^  (2007) | America | observational | 52.8 | 27 | Uncontrolled: 987  (severe or difficult to treat asthma) (GINA) | EG-5D-3L  EQ-VAS | |
| Chen H *et al.*  (2011) | America | observational | 54.4 (9) | 29 | Asthma: 75 | EQ-5D-3L | |
| Chen A *et al.*  (2018) | Canada | observational | 57.78 (17.03) | 35 | Asthma: 537 | HUI-III | |
| Chung *et al.*  (2017) | Korea | observational | 61 (0.2) | 57.9 | Asthma: 5989 | EQ-5D-3L  EQ-VAS | |
| D’Amato *et al.*  (2014) | Italy | experimental | 49.4(13.9) | 50 | Severe: 10  (severe uncontrolled non-apneic asthma) (reversibility >15%) | EQ-5D-3L | |
| Doz *et al.*  (2013) | France, Spain | observational | - France  1. well-controlled: 48.4 (16.6)  2. partly controlled: 49.0 (18.0)  3. uncontrolled: 53.3 (18.3)  - Spain  1. well-controlled: 42.4 (15.9)  2. partly controlled: 45.6 (19.0)  3. uncontrolled: 47.8 (17.7) | - France  1. well-controlled: 45.1  2. partly controlled: 37.8  3. uncontrolled: 339  - Spain  1. well-controlled: 38.7  2. partly controlled: 37.2  3. uncontrolled: 34.2 | - France (GINA)  1. well-controlled: 344  2. partly controlled: 307  3. uncontrolled: 182  - Spain (GINA)  1. well-controlled: 436  2. partly controlled: 498  3. uncontrolled: 529 | EQ-5D-3L  VAS | |
| Ferreira *et al*  (2010) | Portugal | observational | 49.2 (16.9) | 29.8 | Asthma: 115 | SF-6D  EQ-5D-3L  EQ-VAS | |
| Flood *et al.*  (2006) | Italy, France, UK, America | observational | n/a | n/a | Asthma: 251  (intermittent/mild/moderate/severe) (self-reported) | ASUI | |
| Gonzalez-Barcala *et al.*  (2012) | Spain | observational | n/a | n/a | Asthma  (GINA/ACQ)  1. intermittent:520  2. mild:754  3. moderate: 672  4. severe: 87  1. well-controlled: 731  2. partly controlled: 541  3. uncontrolled: 762 | VAS | |
| Gray *et al.*  (2018) | Australia, Canada, Germany, Norway, UK, America | observational | 43.03 (15) | 37.7 | Asthma: 852 | EQ-5D-5L  HUI-III | |
| Hernandez *et al.*  (2018) | France | observational | 30.3 (6.7) | 38.7 | Asthma: 222 | EQ-5D-5L  EQ-VAS | |
| Hernandez *et al.*  (2019) | France, UK | observational | 31 (6.7) | 39.4 | Asthma: 279  (ACQ)  1. well-controlled: 119  2. partly controlled: 82  3. uncontrolled: 66 | EQ-5D-5L  EQ-VAS | |
| Heyworth *et al.*  (2009) | UK | observational | n/a | n/a | Asthma: 429 | EQ-5D-3L  EQ-VAS | |
| Johnson *et al.*  (2019) | Amercia | observational | 48.2 (16.7) | 39.1 | Asthma: 302 | EQ-VAS | |
| Juniper *et al.*  (2001) | Canada | observational | 38 (11) | 30 | Asthma: 40 | SG | |
| Kaambwa *et al.*  *(*2016) | Australia, Canada, Germany, Norway, UK, America | observational | n/a | 38 | Asthma: 856 | EQ-5D-5L  HUI-III  SF-6D  15D | |
| Khan *et al.*  (2018) | Australia, Canada, Germany, Norway, UK, America | observational | n/a | 38 | Asthma: 856 | EQ-5D-5L  TTO  VAS | |
| Kontodimopoulos *et al.*  (2018) | Greece | observational | 46.63 (15.31) | 26.9 | Asthma:104  (GINA/ACQ)  1. mild: 20  2. moderate: 32  3. severe: 52  1. well-controlled: 53  2. partly controlled: 28  3. uncontrolled: 23 | EQ-5D-3L  SF-6D  AQL-5D | |
| Koskela *et al.*  *(*2014) | Finland | observational | 58.3 | 25.7 | Asthma: 996 | 15D | |
| Lanario *et al.*  (2020) | UK | observational | 51 | 37 | Severe: 96  (ERS&ATS guideline) | EQ-VAS | |
| Lubetkin *et al.*  (2005) | America | observational | n/a | n/a | Asthma: 1202 | EQ-5D-3L  EQ-VAS | |
| Lucas *et al.*  (2020) | France | observational | Severe: 42.2 (16.4) | Severe: 42.7 | Severe: 94  (GINA)  Uncontrolled: 57  (ACT) | EQ-5D-5L  EQ-VAS | |
| McTaggart-Cowan *et al*.  (2008) | Canada | observational | 35 (7.9) | 30 | Asthma: 157  (self-reported)  1. mild: 59  2. moderate: 51  3. severe: 25  1. well-controlled: 43  2. partly controlled: 54  3. uncontrolled: 22 | EQ-5D-3L  HUI-III  SF-6D  AQL-5D  EQ-VAS  VAS | |
| Meszaros *et al.*  (2003) | Hungary | experimental | 35.46 | n/a | Asthma: 55 | VAS | |
| Mitchell *et al.*  (2017) | Australia, Canda, UK, America | observational | n/a | 31.1 | Asthma: 579 | EQ-5D-3L  SF-6D  HUI-III  15D  QWB | |
| Mittmann *et al.*  (1999) | Canada | observational | n/a | 39 | Asthma: 1128 | HUI-III | |
| Mittmann *et al.*  (2001) | Canada | observational | n/a | n/a | Asthma: 897 | HUI-III | |
| Moy *et al.*  (2004) | America | observational | 49 (15) | 24 | Asthma: 100  (FEV1)  1. mild: 41  2. moderate: 33  3. severe: 26 | ASUI  HUI-3  RS  SG  TTO | |
| Mungan *et al.*  (2018) | Middle Eastern countries: Egypt, Turkey and a Gulf cluster (Kuwait, Saudi Arabia and United Arab Emirates) | observational | n/a | 42.5 | Asthma: 806  (ACT)  well-controlled: 464 | EQ-5D-3L  EQ-VAS | |
| Peters *et al.*  (2014) | UK | observational | n/a | n/a | Asthma: 255 | EQ-5D-3L  EQ-VAS | |
| Polley *et al.*  (2008) | UK | observational | 51.6 (17.5) | 65 | Asthma: 20 | EQ-5D-3L  EQ-VAS | |
| Retzler *et al.*  (2018) | UK, France, Germany and Slovakia | observational | n/a | n/a | uncontrolled: 710  (GINA) | SG | |
| Revicki *et al.*  (1998) | America | observational | 34.7 (10.7) | 41 | Asthma: 161 | ASUI  HUI-II | |
| Rutten-van Mölken *et al.*  (1995) | Netherlands | experimental | 1. salmeterol: 51 (15)  2. salbutamol: 55 (13) | 1. salmeterol: 50.9  2. salbutamol: 49.1 | Moderate:  (FEV1)  1. salmeterol: 53  2. salbutamol: 54 | SG | |
| Sadatsafavi *et al.*  (2015) | Canada | observational | Overall: 52.2 (14.5)  1. controlled: 53.04 (15.01)  2. partly controlled: 51.41 (14.64)  3. uncontrolled: 52.89 (13.78) | Overall: 33  1. controlled: 40.5  2. partly controlled: 34.3  3. uncontrolled: 24.8 | Asthma: 494  (GINA)  1. well-controlled: 127  2. partly controlled: 194  3. uncontrolled: 173 | AQL-5D  EQ-5D-3L | |
| Smith *et al.*  (2004) | America | observational | 38 (15) | 29.3 | Asthma: 1033 | ASUI | |
| Sullivan *et al.*  (2013) | America | observational | n/a | n/a | Asthma: 1279 | EQ-5D-3L  VAS | |
| Sullivan *et al.*  (2016) | America | observational | 47.9 | 35 | Asthma: 2681 | AQL-5D  EQ-5D-3L  EQ-VAS |  |
| Szende *et al.*  (2004) | Hungary | observational | n/a | n/a | Asthma: 228  (GINA)  1. intermittent: 36  2. mild: 64  3. moderate: 82  4. severe: 46 | EQ-5D-3L  EQ-VAS  SF-6D  TTO | |
| Tarraf *et al.*  (2018) | five Middle Eastern countries (Egypt, Turkey, Kuwait, Saudi Arabia, and the United Arab Emirates, the latter three grouped into a Gulf cluster) | observational | n/a | n/a | Asthma: 2124 | EQ-5D-3L  EQ-VAS | |
| Thomas *et al.*  (2017) | UK | experimental | 57 | 37.4 | Asthma: 261 | EQ-5D-3L | |
| Van der Meer *et al.*  (2011) | Netherlands | experimental | 37 | 29 | Asthma: 99 | EQ-5D-3L  EQ-VAS | |
| Willems *et al.*  (2007) | Netherlands | experimental | 45.9 (15.9) | 33.3 | Asthma: 27 | EQ-5D-3L  EQ-VAS  SF-6D | |
| Wilson *et al.*  (2018) | America | observational | 49.1 (12.3) | 35.4 | Asthma:147 | ASUI | |
| Yong *et al.*  (2016) | Malaysia | observational | n/a | 15 | Asthma: 20 | EQ-5D-3L  EQ-VAS  VAS  SG | |

†Utility values for difficult asthma were considered as utility values for uncontrolled asthma. ACT, Asthma Control Test; ACQ, Asthma Control Questionnaire; ATS, American Thoracic Society; ERS, European Respiratory Society; FEV1, the first second of forced expiration; GINA, The Global Initiative for Asthma

***Supplementary Table 5: Results of quality assessment***

**Supplementary Table S5. Results of quality assessment**

|  |  | Item 1 | Item 2 | Item 3 | Item 4 | Item 5 | Item 6 | Item 7 |
| --- | --- | --- | --- | --- | --- | --- | --- | --- |
|  | Study | Sample size | Respondent  Selection and Recruitment | Inclusion/ Exclusion Criteria | Response Rates | Loss to  Follow-up* | Missing Data | Appropriateness of Measure |
| 1 | Abrunz 2007 | **no** | **yes** | **yes** | **unclear** | **n/a** | **unclear** | **yes** |
| 2 | Al-kalemji 2013 | **yes** | **yes** | **yes** | **yes** | **n/a** | **unclear** | **yes** |
| 3 | Allegra 2012 | **yes** | **yes** | **yes** | **yes** | **n/a** | **unclear** | **yes** |
| 4 | Barton 2008 | **yes** | **unclear** | **yes** | **yes** | **n/a** | **yes** | **yes** |
| 5 | Bime 2012 | **yes** | **unclear** | **yes** | **yes** | **n/a** | **unclear** | **yes** |
| 6 | Blumenschein 1998 | **no** | **no** | **yes** | **yes** | **n/a** | **yes** | **yes** |
| 7 | Burström 2001 | **yes** | **yes** | **unclear** | **yes** | **n/a** | **yes** | **yes** |
| 8 | Chen 2007 | **yes** | **yes** | **yes** | **yes** | **n/a** | **yes** | **yes** |
| 9 | Chen 2011 | **no** | **yes** | **yes** | **yes** | **n/a** | **yes** | **yes** |
| 10 | Chen 2018 | **yes** | **yes** | **yes** | **yes** | **n/a** | **yes** | **yes** |
| 11 | Chung 2017 | **yes** | **yes** | **unclear** | **yes** | **n/a** | **unclear** | **yes** |
| 12 | D' Amato 2014 | **no** | **unclear** | **yes** | **yes** | **yes** | **unclear** | **yes** |
| 13 | Doz 2013 | **yes** | **yes** | **yes** | **yes** | **n/a** | **yes** | **yes** |
| 14 | Ferreira 2010 | **yes** | **unclear** | **yes** | **unclear** | **n/a** | **unclear** | **yes** |
| 15 | Flood 2006 | **no** | **no** | **unclear** | **unclear** | **n/a** | **yes** | **yes** |
| 16 | Gonzalez-Barcala 2012 | **yes** | **yes** | **yes** | **yes** | **n/a** | **unclear** | **yes** |
| 17 | Gray 2018 | **yes** | **yes** | **unclear** | **unclear** | **n/a** | **yes** | **yes** |
| 18 | Hernandez 2018 | **yes** | **yes** | **yes** | **no** | **n/a** | **unclear** | **yes** |
| 19 | Hernandez 2019 | **yes** | **yes** | **yes** | **yes** | **n/a** | **yes** | **yes** |
| 20 | Heyworth 2009 | **yes** | **yes** | **yes** | **no** | **n/a** | **unclear** | **yes** |
| 21 | Johnson 2019 | **yes** | **yes** | **yes** | **yes** | **n/a** | **unclear** | **yes** |
| 22 | Juniper 2001 | **no** | **unclear** | **yes** | **yes** | **n/a** | **unclear** | **yes** |
| 23 | Kaambwa 2016 | **yes** | **yes** | **unclear** | **yes** | **n/a** | **unclear** | **yes** |
| 24 | Khan 2018 | **yes** | **no** | **yes** | **unclear** | **n/a** | **yes** | **yes** |
| 25 | Kontodimopoulos 2018 | **yes** | **yes** | **yes** | **yes** | **n/a** | **unclear** | **yes** |
| 26 | Koskela 2014 | **yes** | **yes** | **yes** | **yes** | **n/a** | **unclear** | **yes** |
| 27 | Lanario 2020 | **no** | **yes** | **yes** | **yes** | **n/a** | **yes** | **yes** |
| 28 | Lubetkin 2005 | **yes** | **yes** | **unclear** | **yes** | **n/a** | **yes** | **yes** |
| 29 | Lucas 2020 | **no** | **yes** | **yes** | **yes** | **n/a** | **yes** | **yes** |
| 30 | McTaggart-Cowan 2008 | **yes** | **yes** | **yes** | **unclear** | **n/a** | **unclear** | **yes** |
| 31 | Meszaros 2003 | **no** | **yes** | **yes** | **yes** | **yes** | **unclear** | **yes** |
| 32 | Mitchell 2017 | **yes** | **yes** | **yes** | **unclear** | **n/a** | **unclear** | **yes** |
| 33 | Mitmann 1999 | **yes** | **yes** | **yes** | **unclear** | **n/a** | **unclear** | **unclear** |
| 34 | Mittmann 2001 | **yes** | **unclear** | **yes** | **yes** | **n/a** | **yes** | **yes** |
| 35 | Moy 2004 | **yes** | **yes** | **yes** | **yes** | **n/a** | **unclear** | **yes** |
| 36 | Mungan 2018 | **yes** | **yes** | **yes** | **yes** | **n/a** | **unclear** | **yes** |
| 37 | Peters 2014 | **yes** | **yes** | **yes** | **yes** | **n/a** | **unclear** | **yes** |
| 38 | Polley 2008 | **no** | **unclear** | **unclear** | **yes** | **n/a** | **unclear** | **yes** |
| 39 | Retzler 2018 | **yes** | **yes** | **unclear** | **yes** | **n/a** | **unclear** | **yes** |
| 40 | Revicki 1998 | **yes** | **unclear** | **yes** | **unclear** | **n/a** | **unclear** | **yes** |
| 41 | Rutten-van Mölken 1995 | **no** | **yes** | **yes** | **yes** | **yes** | **yes** | **yes** |
| 42 | Sadatsafavi 2015 | **yes** | **yes** | **yes** | **yes** | **n/a** | **unclear** | **yes** |
| 43 | Smith 2004 | **yes** | **yes** | **yes** | **yes** | **n/a** | **unclear** | **yes** |
| 44 | Sullivan 2013 | **yes** | **unclear** | **yes** | **yes** | **n/a** | **yes** | **yes** |
| 45 | Sullivan 2016 | **yes** | **yes** | **yes** | **yes** | **n/a** | **unclear** | **yes** |
| 46 | Szende 2004 | **no** | **unclear** | **yes** | **yes** | **n/a** | **unclear** | **yes** |
| 47 | Tarraf 2018 | **yes** | **yes** | **yes** | **no** | **n/a** | **unclear** | **yes** |
| 48 | Thomas 2017 | **yes** | **yes** | **yes** | **no** | **yes** | **yes** | **yes** |
| 49 | Van der Meer 2011 | **no** | **unclear** | **yes** | **yes** | **yes** | **yes** | **yes** |
| 50 | Willems 2007 | **no** | **unclear** | **yes** | **yes** | **yes** | **yes** | **yes** |
| 51 | Wilson 2018 | **yes** | **yes** | **yes** | **yes** | **n/a** | **unclear** | **yes** |
| 52 | Yong 2016 | **no** | **no** | **no** | **yes** | **n/a** | **yes** | **yes** |

* Only for experimental studies

***Supplementary Figure 1: Forest plots of asthma utility representing asthma***


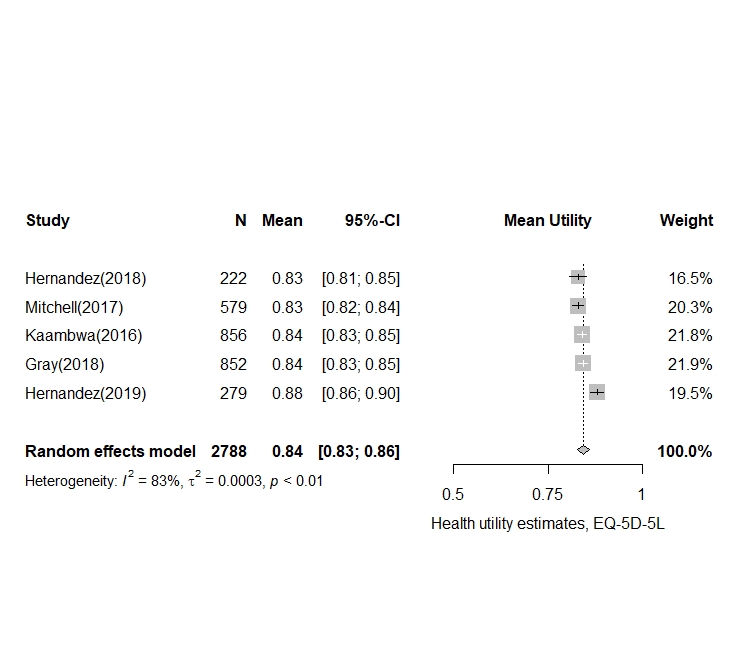


**Supplementary Figure S1.1. Forest plot of asthma utility, using the EQ-5D-5L instrument**


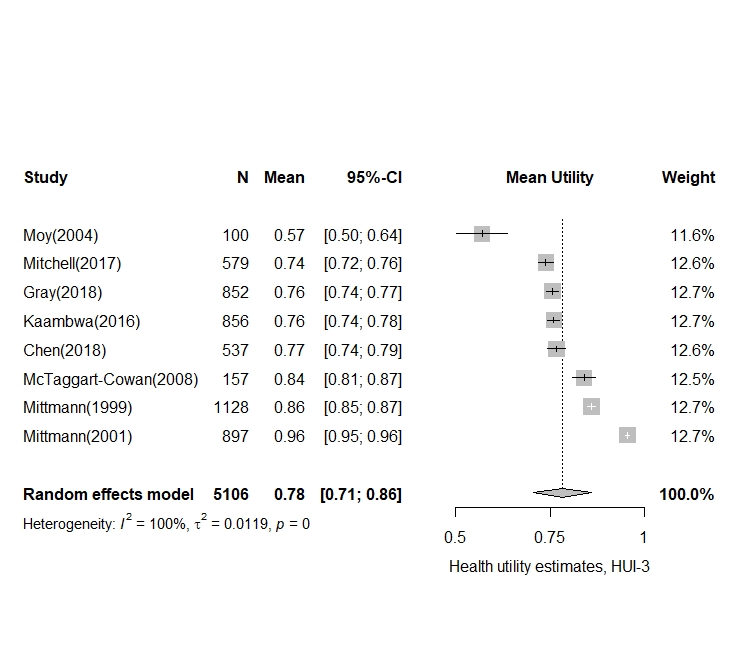


**Supplementary Figure S1.2. Forest plot of asthma utility, using the HUI-3 instrument**


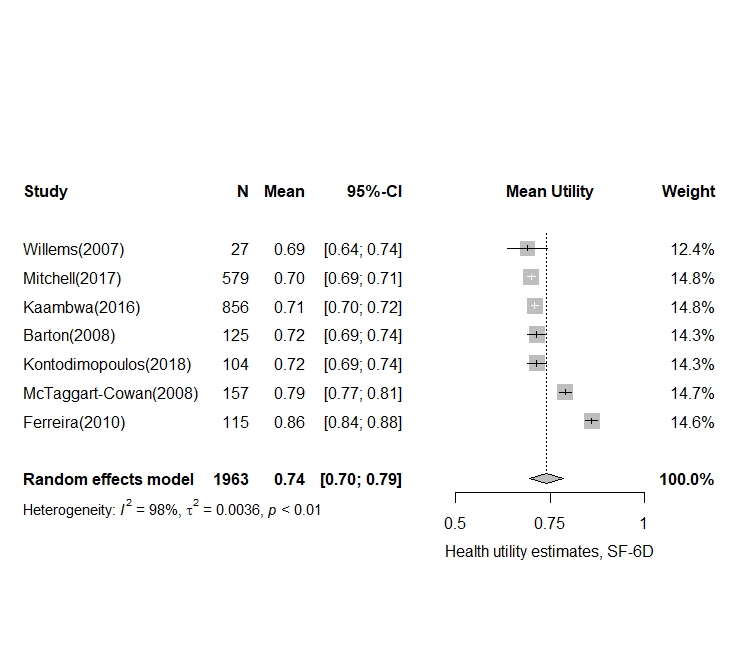


**Supplementary Figure S1.3. Forest plot of asthma utility, using the SF-6D instrument**

**
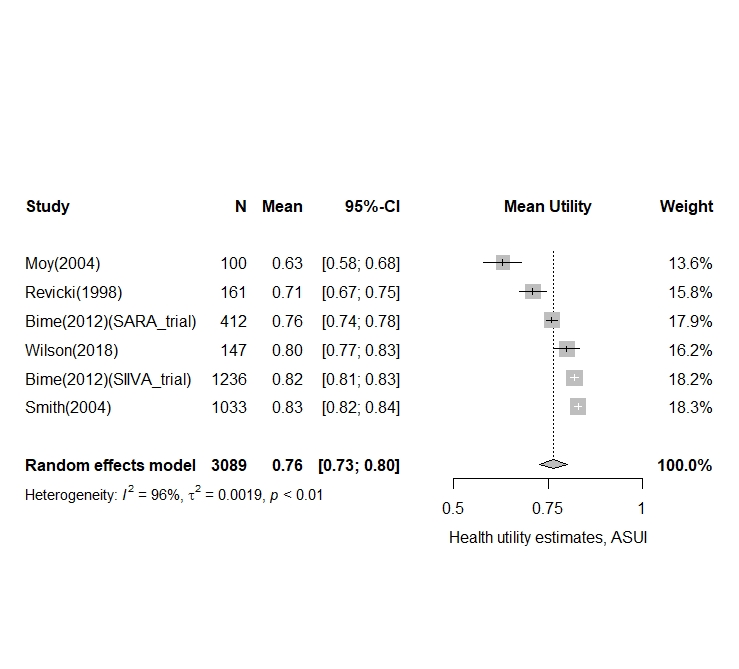
**

**Supplementary Figure S1.4. Forest plot of asthma utility, using the ASUI instrument**


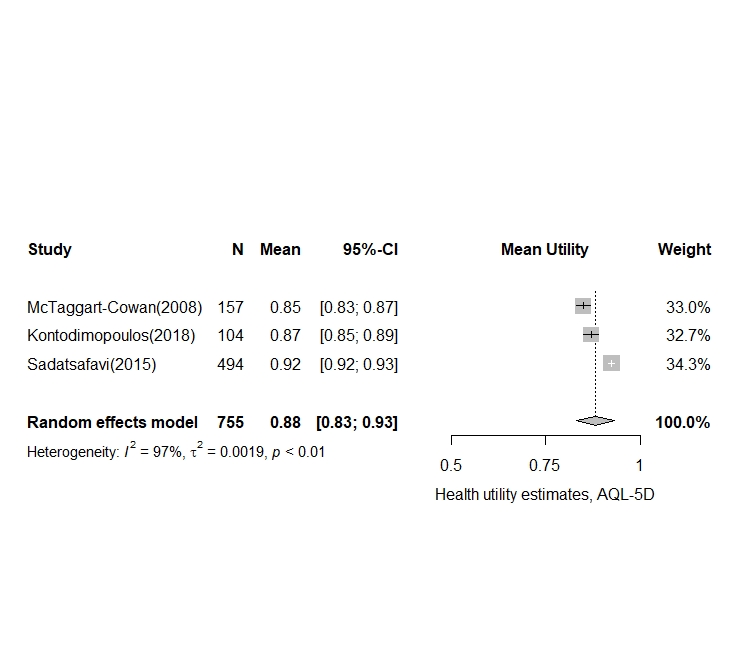


**Supplementary Figure S1.5. Forest plot of asthma utility, using the AQL-5D instrument**

**
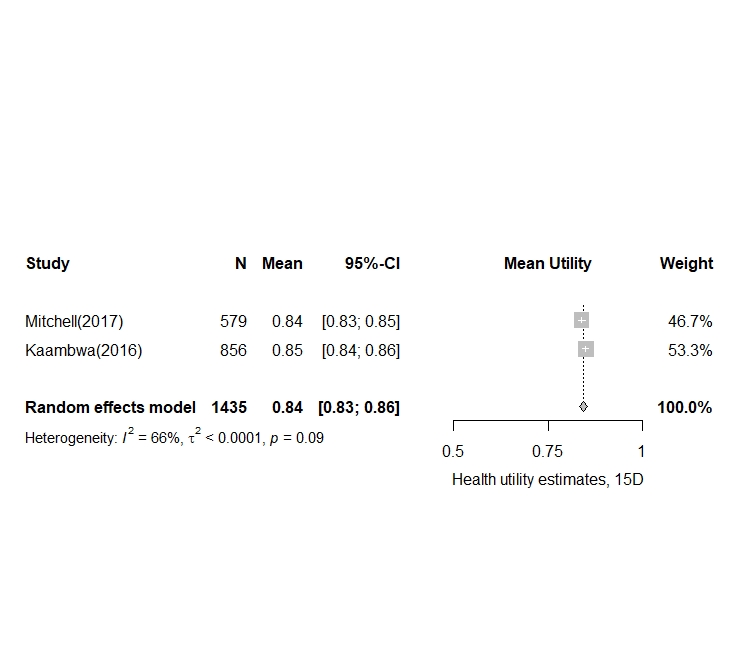
**

**Supplementary Figure S1.6. Forest plot of asthma utility, using the 15D instrument**


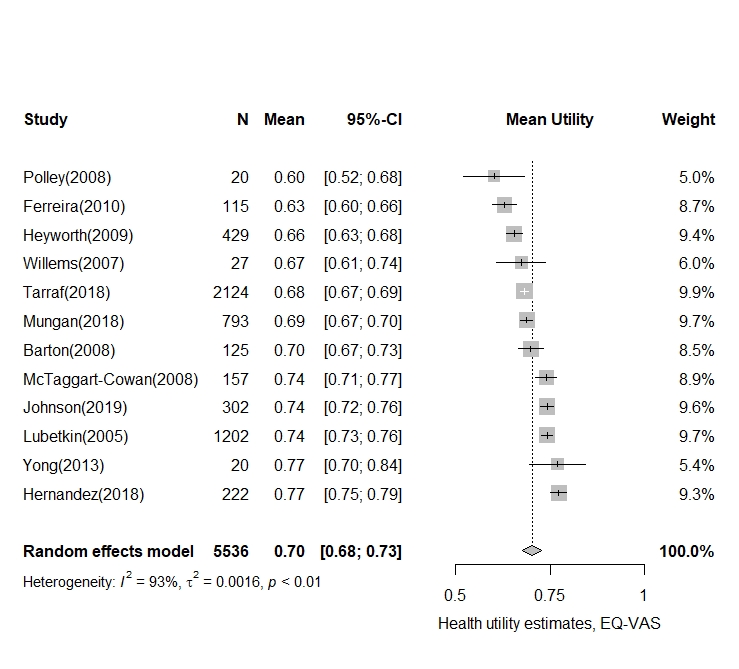


**Supplementary Figure S1.7. Forest plot of asthma utility, using the EQ-VAS instrument**

**
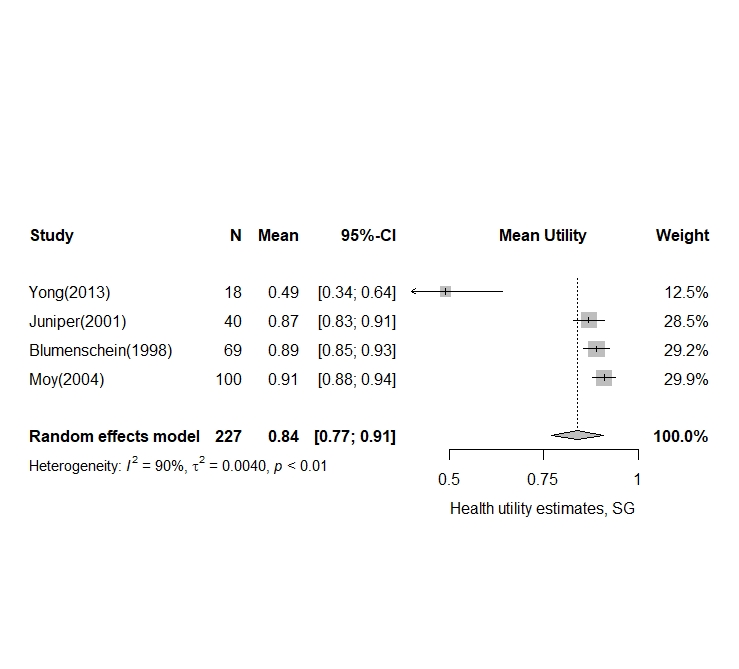
**

**Supplementary Figure S1.8. Forest plot of asthma utility, using the SG instrument**

**
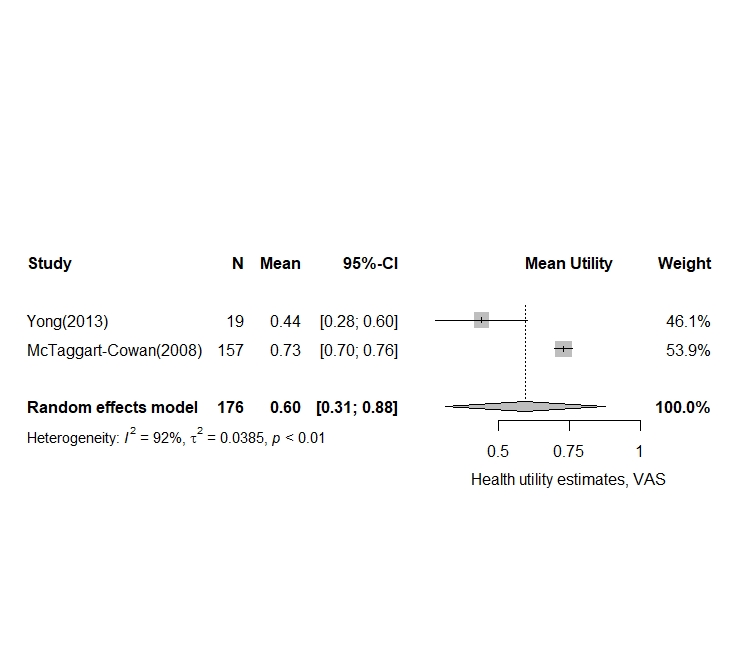
**

**Supplementary Figure S1.9. Forest plot of asthma utility, using the VAS instrument**

**
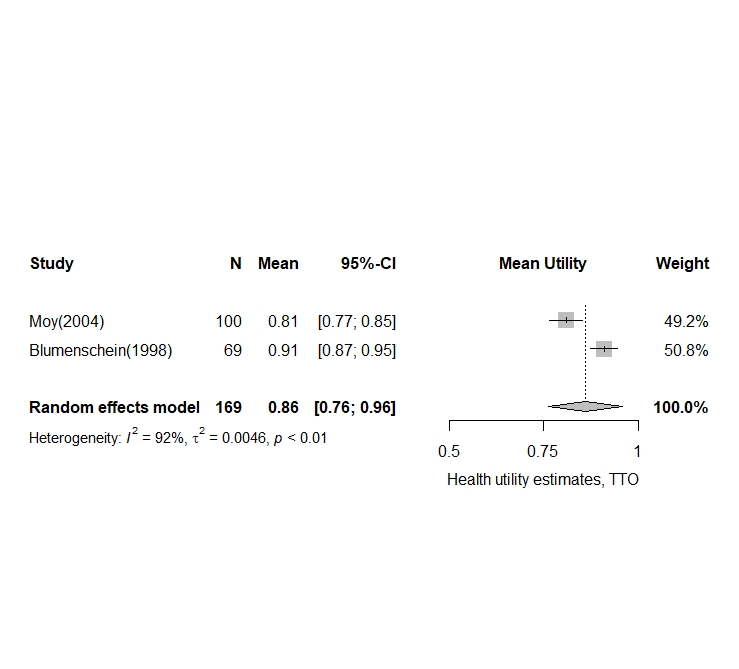
**

**Supplementary Figure S1.10. Forest plot of asthma utility, using the TTO instrument**

***Supplementary Figure 2: Forest plots for each utility instrument stratified by severity and control level***

**
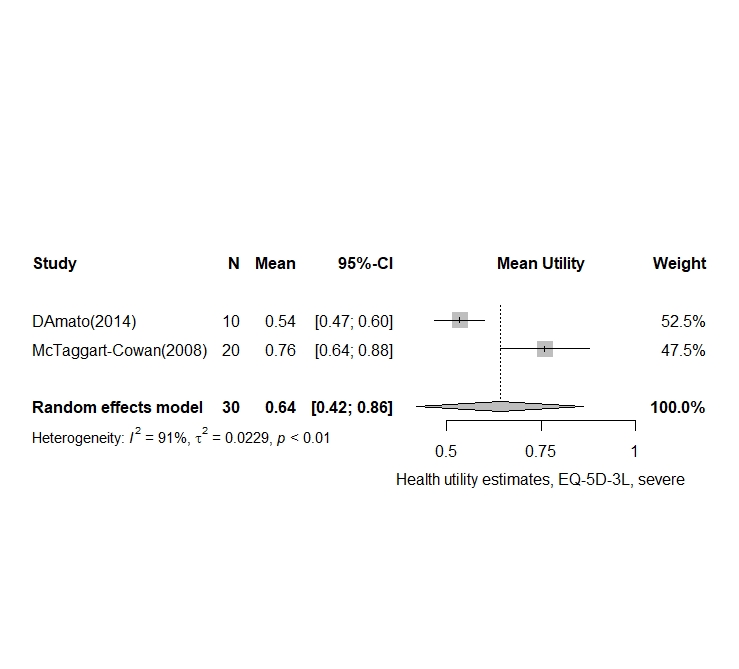
**

**Supplementary Figure S2.1. Forest plot of severe asthma utility, using the EQ-5D-3L instrument**

**
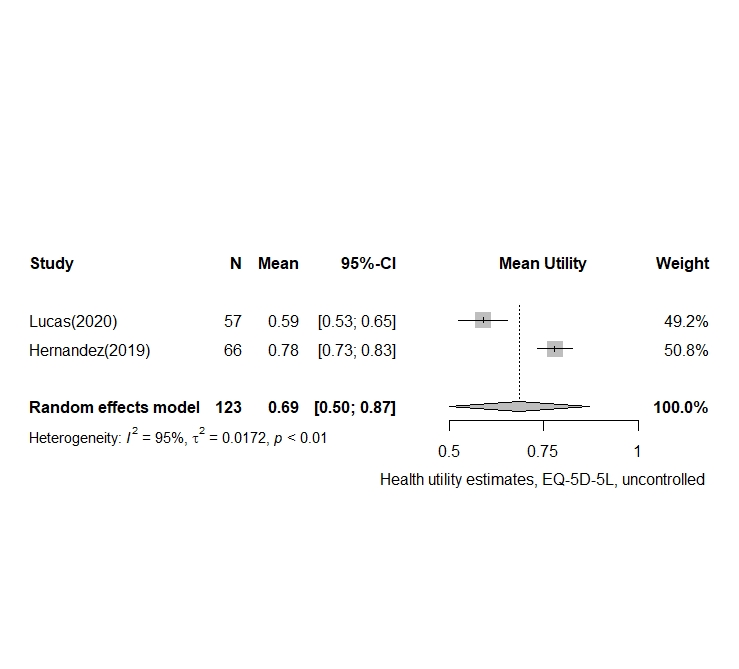
**

**Supplementary Figure S2.2. Forest plot of uncontrolled asthma utility, using the EQ-5D-5L instrument**


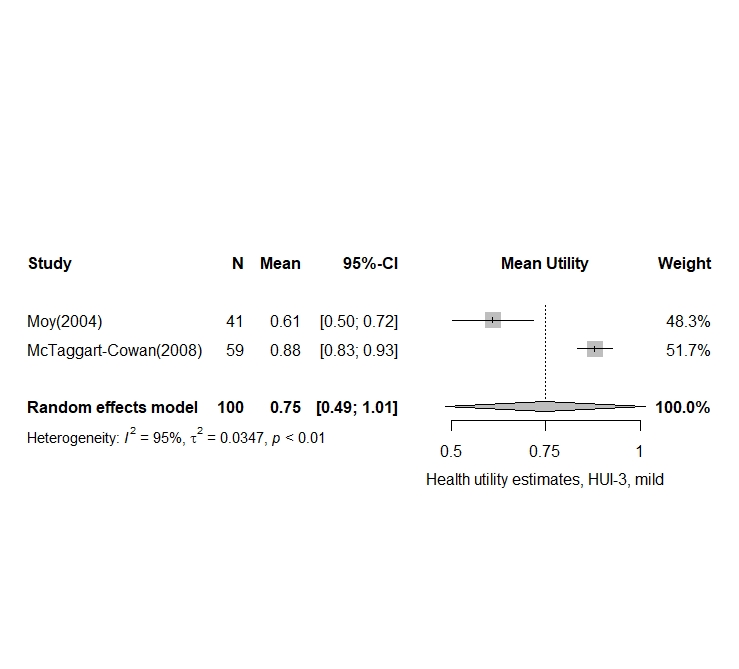


**Supplementary Figure S2.3. Forest plot of mild asthma utility, using the HUI-3 instrument**

**
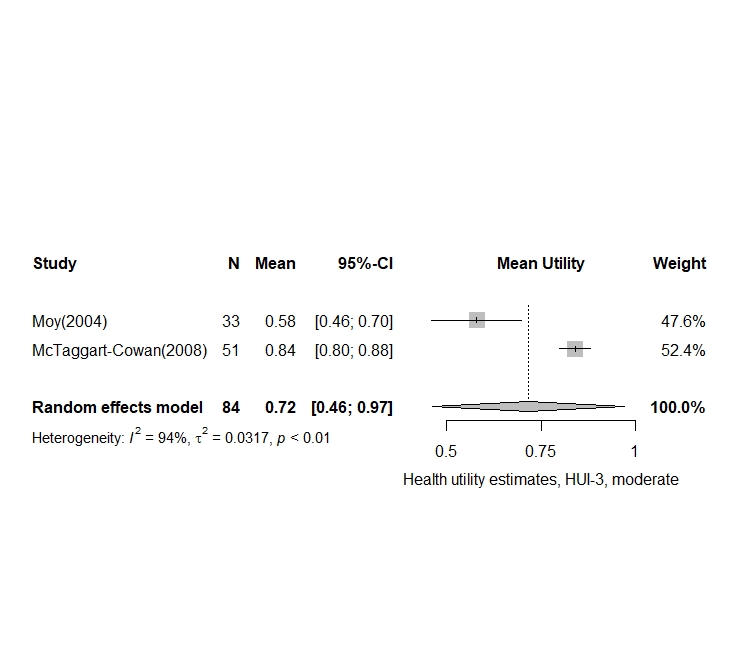
**

**Supplementary Figure S2.4. Forest plot of moderate asthma utility, using the HUI-3 instrument**


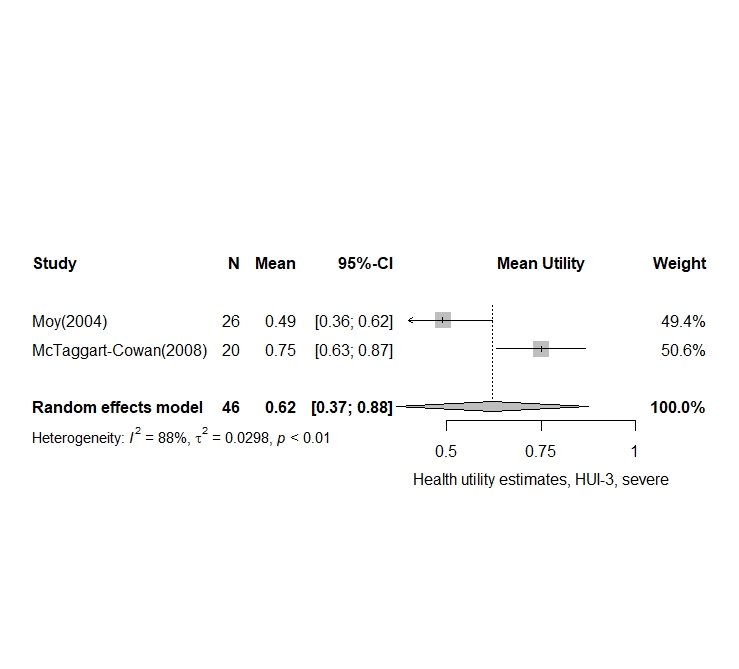


**Supplementary Figure S2.5. Forest plot of severe asthma utility, using the HUI-3 instrument**


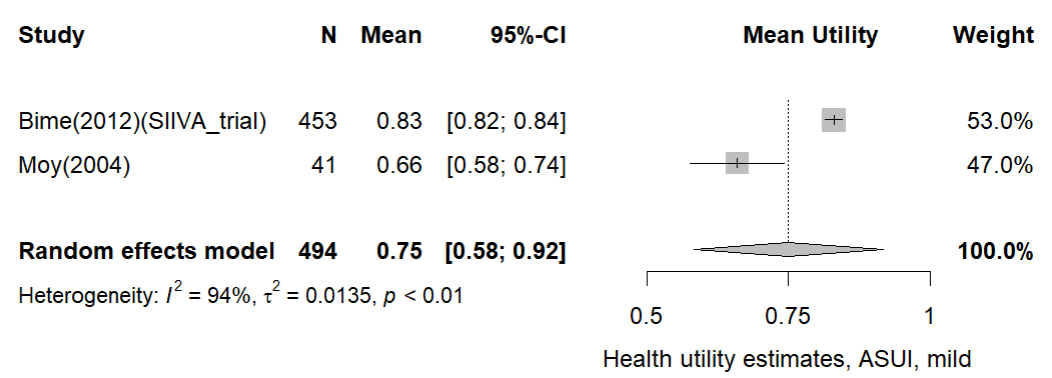


**Supplementary Figure S2.6. Forest plot of mild asthma utility, using the ASUI instrument**


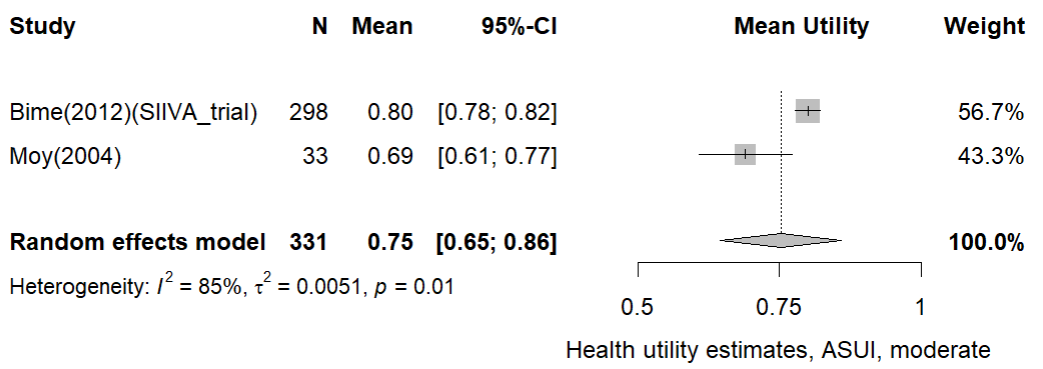


**Supplementary Figure S2.7. Forest plot of moderate asthma utility, using the ASUI instrument**


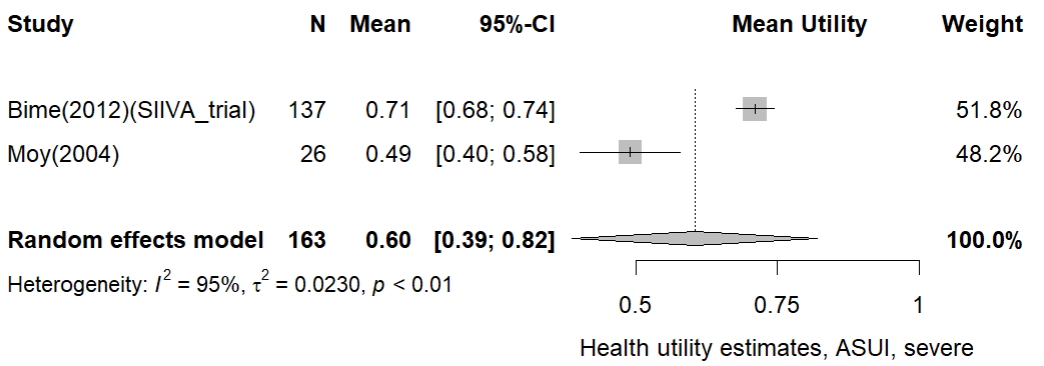


**Supplementary Figure S2.8. Forest plot of severe asthma utility, using the ASUI instrument**


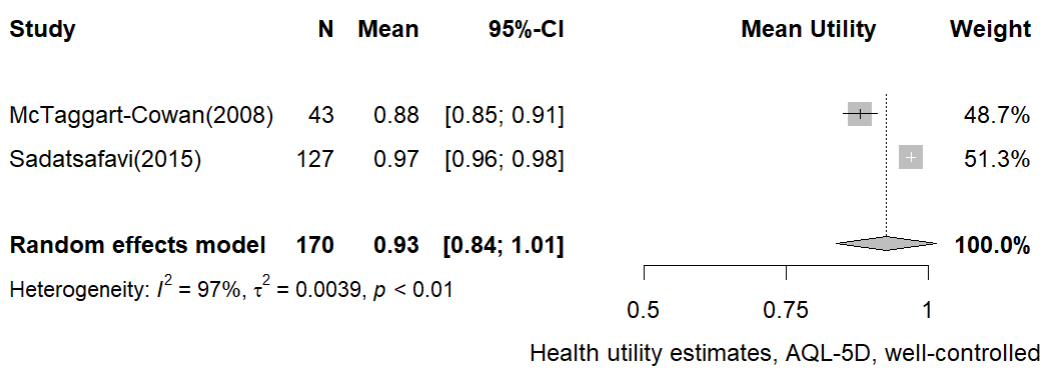


**Supplementary Figure S2.9. Forest plot of well-controlled asthma utility, using the AQL-5D instrument**


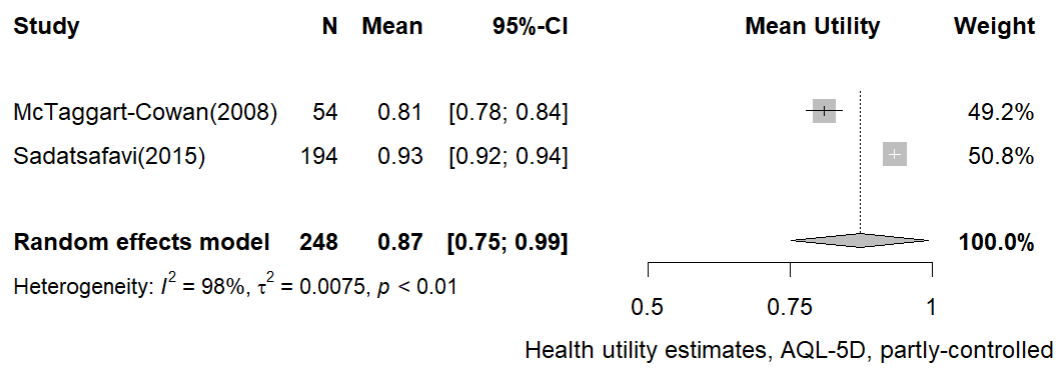


**Supplementary Figure S2.10. Forest plot of partly-controlled asthma utility, using the AQL-5D instrument**


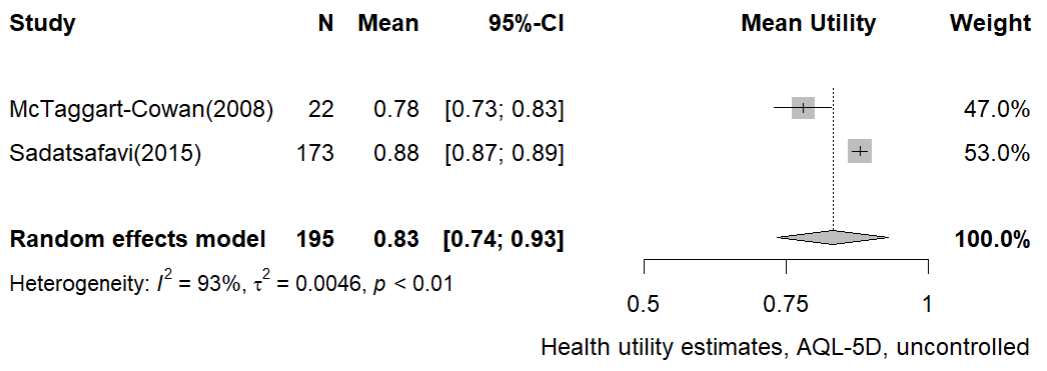


**Supplementary Figure S2.11. Forest plot of uncontrolled asthma utility, using the AQL-5D instrument**

***Supplementary Figure 3: Funnel plots***


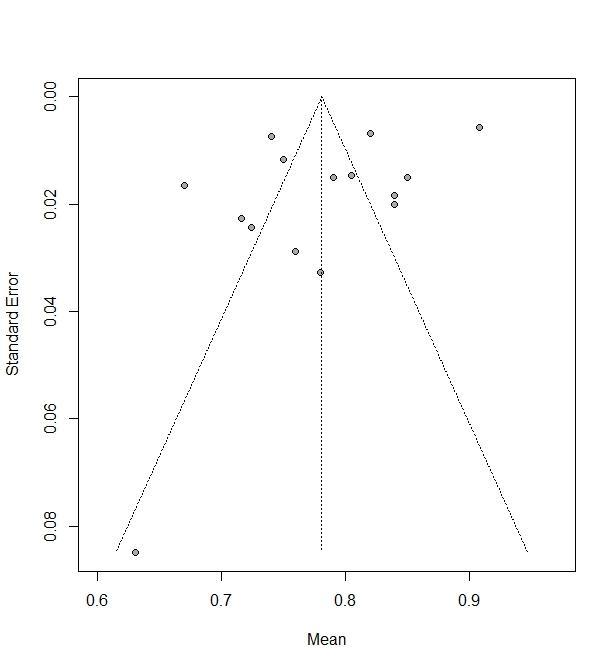


Egger’s regression test for funnel plot asymmetry: t=-1.78, p-value=0.10

**Supplementary Figure S3.1. Funnel plot for the EQ-5D-3L instrument**


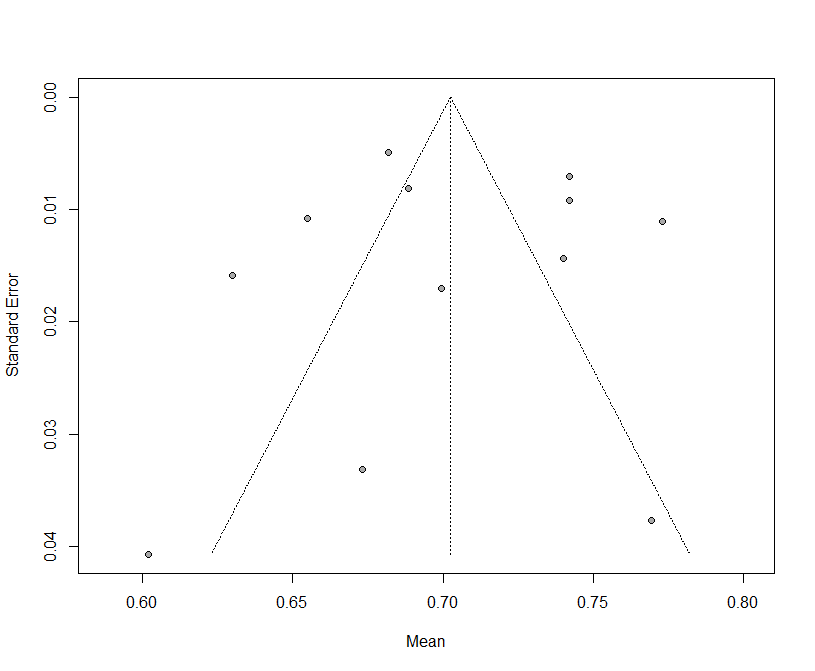


Egger’s regression test for funnel plot asymmetry: t=0.05, p-value=0.96

**Supplementary Figure S3.2. Funnel plot for the EQ-VAS instrument**

***Supplementary Figure 4: Sensitivity analysis excluding studies with unspecified control level criteria***


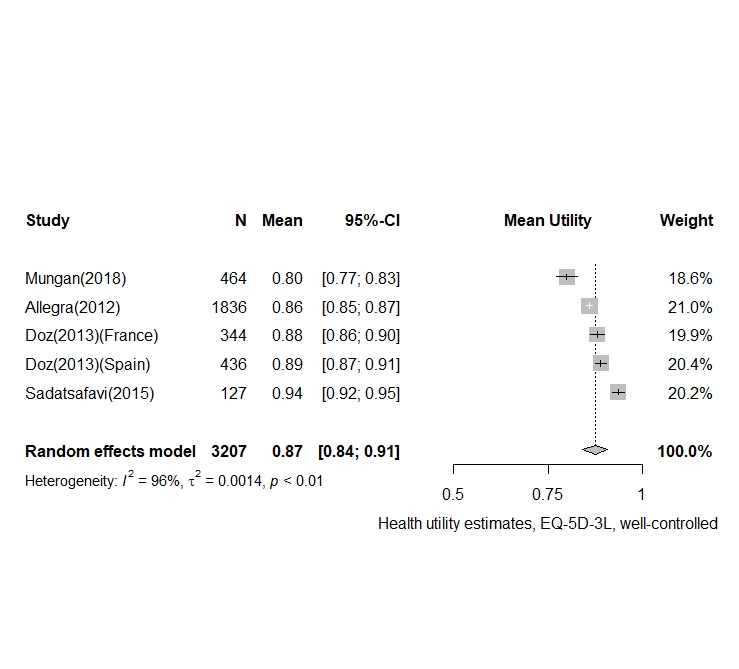


**Supplementary Figure S4.1. Forest plot of well-controlled asthma utility, using the EQ-5D-3L instrument**


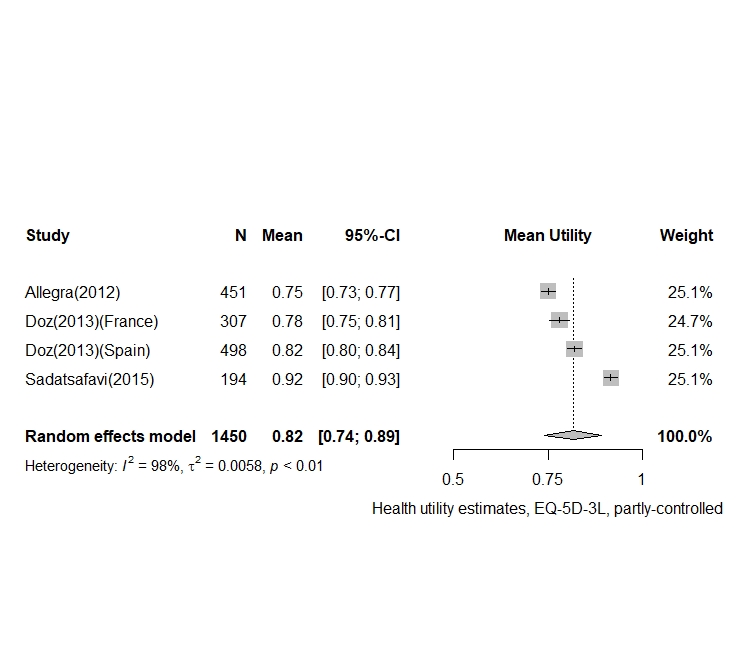


**Supplementary Figure S4.2. Forest plot of partly controlled asthma utility, using the EQ-5D-3L instrument**


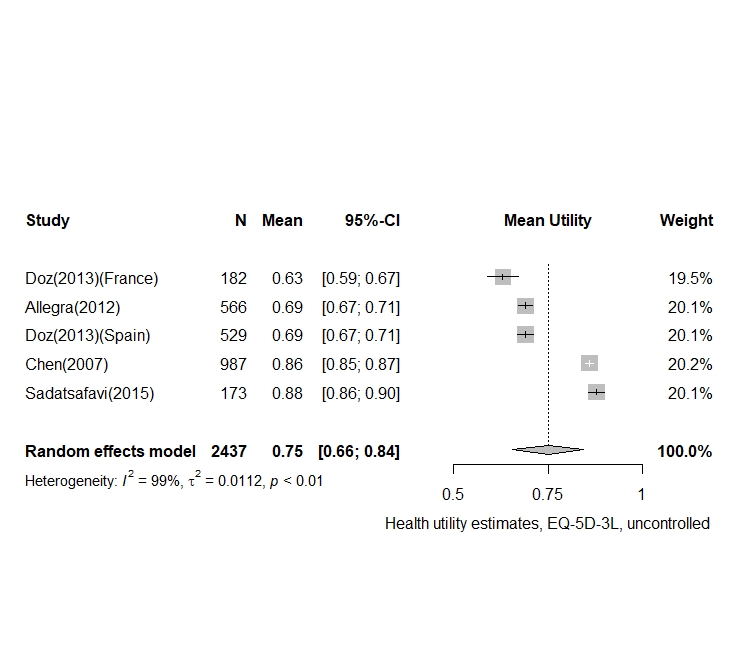


**Supplementary Figure S4.3. Forest plot of uncontrolled asthma utility, using the EQ-5D-3L instrument**

***Supplementary Figure 5: Sensitivity analysis using the leave-one-out method***


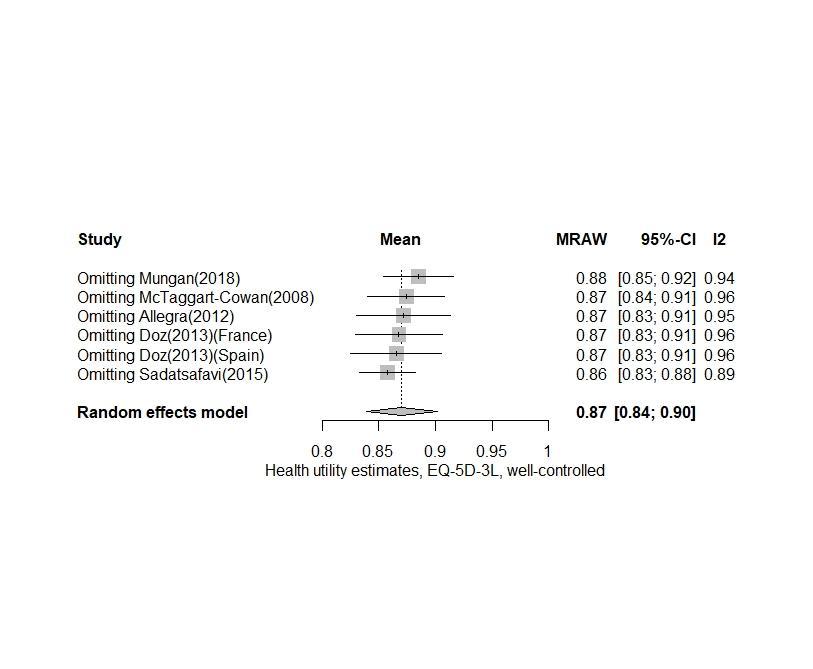


**Supplementary Figure S5.1. Forest plot of well-controlled asthma utility, using the EQ-5D-3L instrument**


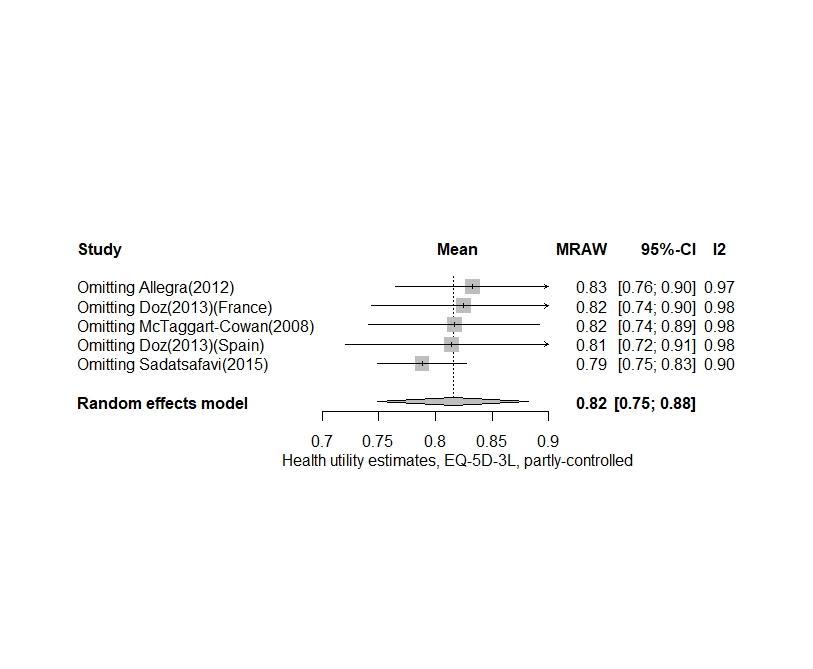


**Supplementary Figure S5.2. Forest plot of partly controlled asthma utility, using the EQ-5D-3L instrument**


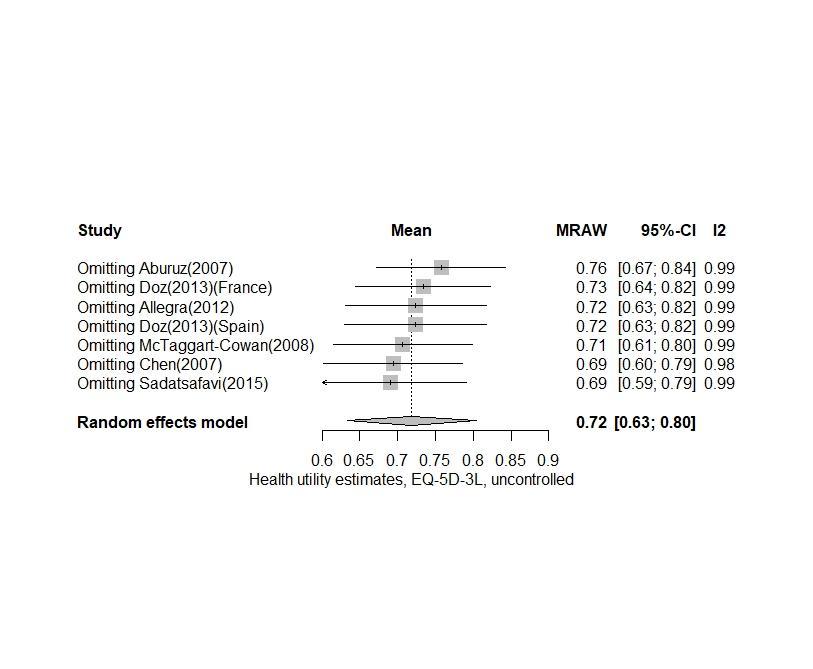


**Supplementary Figure S5.3. Forest plot of uncontrolled asthma utility, using the EQ-5D-3L instrument**
